# The conserved LEM-3/Ankle1 nuclease is involved in the combinatorial regulation of meiotic recombination repair and chromosome segregation in *Caenorhabditis elegans*

**DOI:** 10.1371/journal.pgen.1007453

**Published:** 2018-06-07

**Authors:** Ye Hong, Maria Velkova, Nicola Silva, Marlène Jagut, Viktor Scheidt, Karim Labib, Verena Jantsch, Anton Gartner

**Affiliations:** 1 Centre for Gene Regulation and Expression, University of Dundee, Dundee, United Kingdom; 2 MRC Protein Phosphorylation and Ubiquitylation Unit, School of Life Sciences, University of Dundee, Dundee, United Kingdom; 3 Department of Chromosome Biology, Max F. Perutz Laboratories, University of Vienna, Vienna BioCenter, Austria; University of California, Davis, UNITED STATES

## Abstract

Homologous recombination is essential for crossover (CO) formation and accurate chromosome segregation during meiosis. It is of considerable importance to work out how recombination intermediates are processed, leading to CO and non-crossover (NCO) outcome. Genetic analysis in budding yeast and *Caenorhabditis elegans* indicates that the processing of meiotic recombination intermediates involves a combination of nucleases and DNA repair enzymes. We previously reported that in *C*. *elegans* meiotic joint molecule resolution is mediated by two redundant pathways, conferred by the SLX-1 and MUS-81 nucleases, and by the HIM-6 Bloom helicase in conjunction with the XPF-1 endonuclease, respectively. Both pathways require the scaffold protein SLX-4. However, in the absence of all these enzymes, residual processing of meiotic recombination intermediates still occurs and CO formation is reduced but not abolished. Here we show that the LEM-3 nuclease, mutation of which by itself does not have an overt meiotic phenotype, genetically interacts with *slx-1* and *mus-81* mutants, the respective double mutants displaying 100% embryonic lethality. The combined loss of LEM-3 and MUS-81 leads to altered processing of recombination intermediates, a delayed disassembly of foci associated with CO designated sites, and the formation of univalents linked by SPO-11 dependent chromatin bridges (dissociated bivalents). However, LEM-3 foci do not colocalize with ZHP-3, a marker that congresses into CO designated sites. In addition, neither CO frequency nor distribution is altered in *lem-3* single mutants or in combination with *mus-81* or *slx-4* mutations. Finally, we found persistent chromatin bridges during meiotic divisions in *lem-3; slx-4* double mutants. Supported by the localization of LEM-3 between dividing meiotic nuclei, this data suggest that LEM-3 is able to process erroneous recombination intermediates that persist into the second meiotic division.

## Author summary

Meiotic recombination is required for genetic diversity and for proper chromosome segregation. Recombination intermediates, such as Holliday junctions (HJs), are generated and eventually resolved to produce crossover (CO) and non-crossover (NCO). While an excess of meiotic double-strand breaks is generated, most breaks are repaired without leading to a CO outcome and usually only one break for each chromosome pair matures into a CO-designated site in *Caenorhabditis elegans*. The resolution of meiotic recombination intermediates and CO formation have been reported to be highly regulated by several structure-specific endonucleases and the Bloom helicase. However, little is known about the enzymes involved in the NCO recombination intermediate resolution. In this study, we found that a conserved nuclease LEM-3/Ankle1 acts in parallel to the SLX-1/MUS-81 pathway to process meiotic recombination intermediates. Mutation of *lem-3* has no effect on CO frequency and distribution. Interestingly, prominent accumulation of LEM-3 is found between dividing meiotic nuclei. We provide evidence that LEM-3 is also involved in processing remaining, erroneous recombination intermediates during meiotic divisions.

## Introduction

Meiosis is comprised of two specialized cell divisions that elicit the reduction of the diploid genome to haploid gametes. Homologous recombination occurs in the first meiotic division and is required for meiotic crossover (CO) formation [1]. COs are needed to shuffle genetic information between maternal and paternal chromosomes and are thus required to ensure genetic diversity. COs become cytologically visible as chiasmata and also provide stable connections between maternal and paternal homologous chromosomes (homologues). Chiasmata counter the spindle force and thereby facilitate the accurate segregation of homologues in the first meiotic division.

Meiotic recombination is initiated by DNA double-strand breaks (DSBs) generated by the conserved meiosis-specific Spo11 protein [2]. The number of DSBs generated by Spo11 exceeds the number of COs, ranging from ~2:1 in *Saccharomyces cerevisiae* to ~20:1 in maize [3–6]. In *Caenorhabditis elegans*, each chromosome pair receives 4–7 DSBs over the course of prophase I and typically only one DSB per homologous pair will mature into a CO event [7, 8]. It is thought that the excessive number of DSBs is required to ensure that at least one CO occurs on each homologue, a notion supported by checkpoint mechanisms that delay meiotic prophase progression when the number of DSBs is reduced [9–11]. It is unclear how the obligate CO is selected from the pool of DSBs. The CO selection (or designation) correlates with the congression of several pro-CO factors into six distinct foci, one on each paired chromosome starting from the mid-pachytene stage. These include the cyclin-related protein COSA-1/CNTD1, MSH-4/MSH-5 components of the MutSγ complex, the predicted ubiquitin ligases ZHP-3/RNF212 and HEI10, the Bloom (BLM) helicase HIM-6 as well as its regulatory subunit RMH-1 [12–17].

When the CO designation sites are associated with those pro-CO factors, processing of meiotic recombination intermediates is biased towards the CO outcome. One of the meiotic recombination intermediates is called a Holliday junction (HJ), a cruciform DNA structure formed as a result of a reciprocal exchange of DNA strands between homologous chromosomes [18]. While in fission yeast single HJs appear to predominate, direct evidence for the occurrence of double HJs (dHJs) was obtained in budding yeast [19]. dHJs can be processed to result in CO or a non-crossover (NCO) outcome, depending on the directionality of the cut made by structure-specific endonucleases [20]. There is emerging evidence that a combination of nucleases is required for the processing of meiotic HJs to promote CO formation and to resolve joint DNA structures that might impede proper chromosome segregation [21]. Only in fission yeast, deletion of a single nuclease MUS81, leads to a defect in meiotic CO formation [22]. In budding yeast, absence of the MUS81-MMS4, SLX1-SLX4 or YEN1 nucleases exhibits a modest reduction of meiotic COs [23]. The Exo1 nuclease and the mismatch-repair MutLγ complex Mlh1-Mlh3 have also been shown to contribute to HJ resolution [23, 24]. Mouse *gen1* mutants have no meiotic phenotype, while *mus81* animals only show minor defects [25]. In *C*. *elegans* HJ resolution and CO formation appear to be conferred by at least two redundant pathways [26–28]. One pathway is defined by the MUS-81 and SLX-1 nucleases. Consistent with *in vitro* nuclease assays, it appears that SLX-1 might confer a first nick on a HJ, the nicked HJ being the preferred substrate for MUS-81 [29]. The second pathway comprises the XPF-1 nuclease and the BLM helicase HIM-6. It is possible that HIM-6 might be able to unwind a HJ, which would generate a substrate cleaved by XPF-1. Both pathways require SLX-4 as a scaffold protein. When both pathways are compromised, the CO frequency is reduced by roughly one third [26].

Since only a small subset of DSBs are designated as CO sites, the majority of DSBs have to be processed to favour inter-homolog NCO and/ or inter-sister recombination [30]. In budding yeast recombination events leading to the majority of NCO events mature early, whereas the CO events mature later [31, 32]. Several helicases are proposed to mediate the disassembly of early recombination intermediates such as D-loop structures in a pathway called synthesis-dependent strand annealing (SDSA). In budding yeast, this is driven by the Srs2 helicase [33]. In animals, this activity is ascribed to the BLM and RTEL helicases. In *C*. *elegans* deletion of the RTEL-1 helicase leads to an elevated number of meiotic COs [34]. In contrast, deletion of *him-6*, the *C*. *elegans* BLM homologue, leads to reduced meiotic CO formation [34]. The presence of HIM-6 at CO designation sites infers a late pro-CO function [35, 36]. Once dHJs are formed, they can either be dissolved by the BLM helicase and Top3 topoisomerase in a NCO manner or resolved by nucleases to form CO or NCO [33].

In this study, we report on roles of the LEM-3/Ankle1 nuclease in processing meiotic recombination intermediates. LEM-3 is only conserved in animals and the mammalian ortholog is referred to as Ankle1 [37, 38]. *C*. *elegans lem-3* mutants are hypersensitive to ionizing irradiation, UV treatment and DNA cross-linking agents [37]. LEM-3/Ankle1 contains an N-terminal LEM domain, Ankyrin repeats and a GIY-YIG nuclease motif. The same nuclease motif can also be found in bacterial UvrC nucleotide excision repair proteins and in the distantly related SLX1 nuclease [39]. Our data show that LEM-3 and MUS-81 act in conjunction to process early recombination intermediates in meiosis. Loss of LEM-3 and MUS-81 leads to aberrant profiles of recombination markers, delayed processing of markers for CO designation, increased apoptotic cell death and the formation of dissociated bivalents. In addition, we found that a considerable pool of LEM-3 localizes between dividing meiotic nuclei and chromosome segregation is compromised due to persistent chromatin linkages in the absence of both LEM-3 and SLX-4, indicating that LEM-3 is able to process erroneous recombination intermediates that persist into meiotic divisions.

## Results

### LEM-3 acts in a genetic pathway parallel to SLX-4 and MUS-81/SLX-1

**Fig 1:**
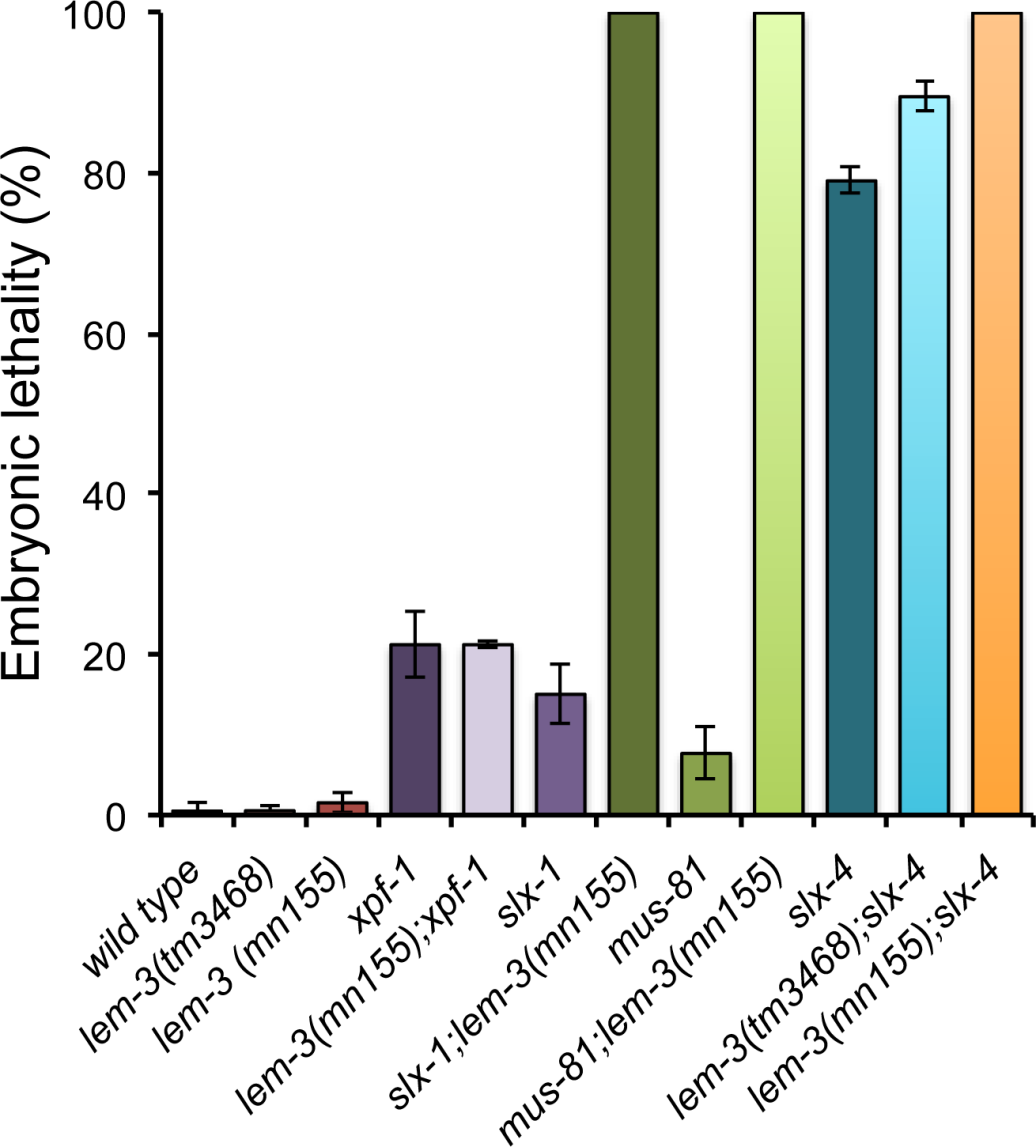
Genetic interaction between LEM-3, MUS-81, SLX-1 and SLX-4 endonucleases. Embryonic lethality in % was determined by counting number of dead eggs/total number of eggs laid. Error bars represent standard deviation of the mean. Sample sizes of indicated genotype are as follows: wild type n = 328, *lem-3 (tm3468)* n = 527, *lem-3 (mn155)* n = 357, *xpf-1* n = 269, *lem-3 (mn155); xpf-1* n = 264, *slx-1* n = 143, *slx-1; lem-3 (mn155)* n = 210, *mus-81* n = 181, *mus-81 lem-3 (mn155)* n = 250, *slx-4* n = 377, *lem-3 (tm3468); slx-4* n = 407, *lem-3 (mn155); slx-4* n = 233.

We and other groups previously showed that there are at least two pathways needed for the resolution of meiotic recombination intermediates: one dependent on SLX-1—MUS-81 and the other relying on XPF-1. SLX-4 acts as a scaffold component in both pathways [26–28]. Given that viability and CO recombination are reduced but not eliminated when both the MUS-81 and XPF-1 pathways are compromised, we considered that there might be at least one additional nuclease that had not been identified. We therefore searched for nucleases which are synthetic lethal with SLX-4 and focused on LEM-3 in this study. Out of the three previously reported LEM-3 alleles, we used the *lem-3 (mn155)* and the *lem-3 (tm3468)*, the former leads to a premature stop codon at amino acid 190 leaving the N-terminal Ankyrin Repeat domain intact, but eliminating the nuclease domain, thus representing a null allele [37]. *lem-3 (tm3468)* bears an in-frame deletion of 110 amino acids between the Ankyrin Repeat and the LEM domain [37]. We found that the lethality of *lem-3 (tm3468); slx-4* was increased to 90% while *lem-3 (mn155); slx-4* worms were 100% embryonic lethal (Fig 1). We employed *lem-3 (mn155)* for our genetic analysis since it is a null allele for *lem-3*, and generated double mutants with *slx-1*, *mus-81 and xpf-1*: 100% of eggs laid by the *slx-1 lem-3* double mutants failed to develop. 100% embryonic lethality was also observed in broods laid by *mus-81 lem-3* double mutants (Fig 1). In contrast, the lethality of *lem-3; xpf-1* double mutants was not different from *xpf-1* mutants (Fig 1). In summary, these genetic data support two potential roles for LEM-3 to maintain embryonic viability: one parallel to SLX-4 and another parallel to MUS-81 and SLX-1.

### Meiotic chromosome axis formation is normal in *lem-3* mutants

We next wanted to test if the synthetic phenotypes we observed were linked to defects in meiotic chromosome axis formation, which plays a central role in organization and dynamics of meiotic chromosomes. In *C*. *elegans*, meiotic prophase progression, which occurs in a gradient of differentiation, can be visualized using dissected germlines. At the distal end of the gonad germ cell mitotically divide, before entering the transition zone where meiotic chromosomes reorganise into arrays of chromatin loops anchored to the chromosome axis [40]. The chromosome axis establishes a platform for homologous chromosome paring, DSB induction, synaptonemal complex assembly, and CO formation [41]. Highlighting the axial element HTP-3 indicated that chromosome axis formation occurred normally in *lem-3* and *slx-4* single mutants and *lem-3; slx-4* double mutants (S1 Fig).

### LEM-3 is required for processing of meiotic recombination intermediates

**Fig 2:**
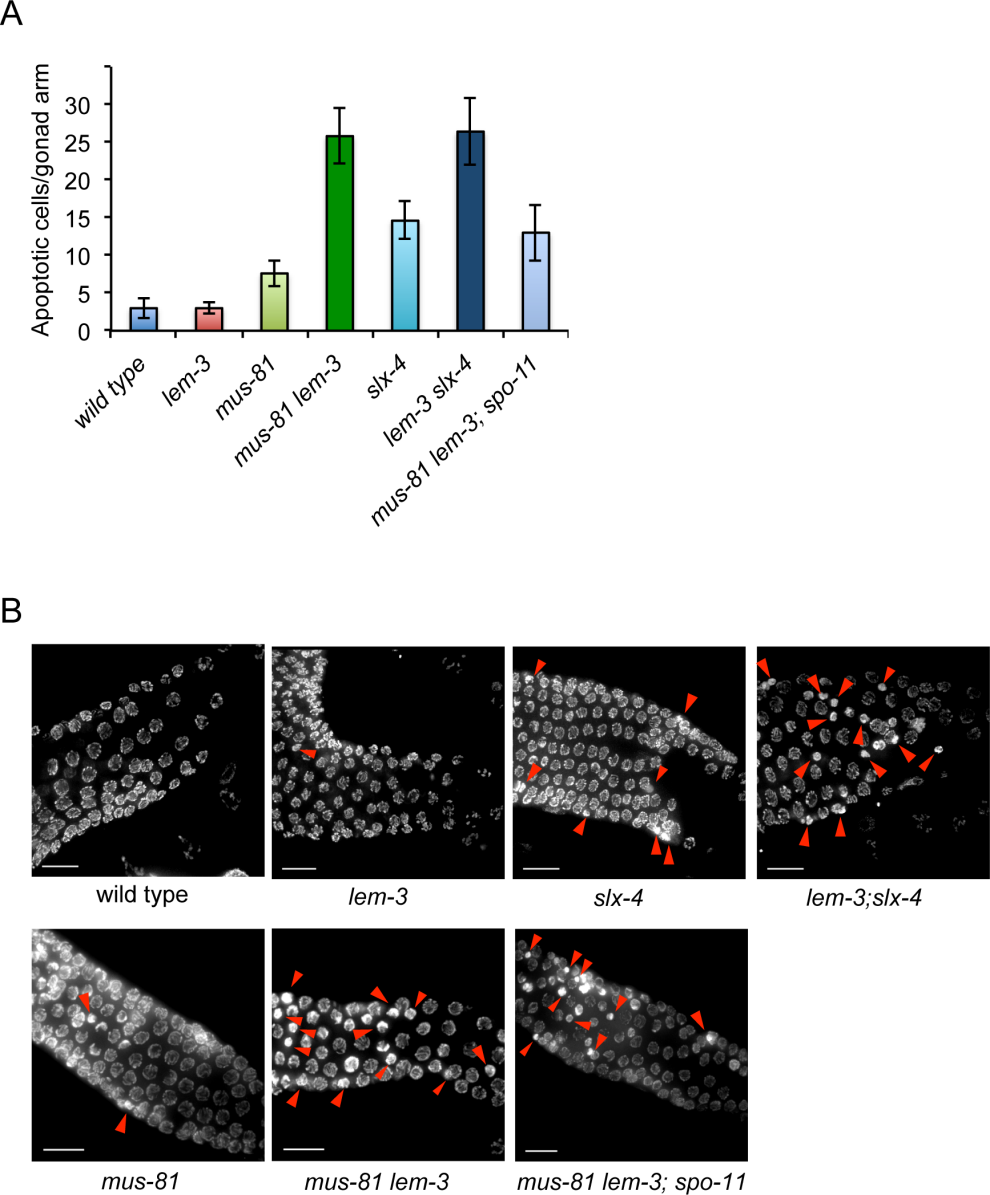
Mutation of *lem-3* in *mus-81* and *slx-4* mutants causes increased apoptosis. (A) Quantification of apoptotic cells per gonad in the indicated genotypes. The apoptotic cells were measured using DIC microscopy. N = 5 gonad arms for each genotype. Error bars represent standard deviation of the mean. (B) Representative images of DAPI-stained germline in wild type, *lem-3*, *mus-81*, *slx-4*, *mus-81 lem-3*, *lem-3; slx-4* and *mus-81 lem-3; spo-11* mutants. Red arrowheads indicate pyknotic cells with abnormally condensed nuclei in the pachytene stage. Scale bars: 15 μm.

Since there are no overt defects in chromosome axis formation, we assessed whether the synthetic lethality we observed was due to a defect in meiotic recombination. In *C*. *elegans*, unrepaired DSBs activate checkpoints that induce apoptosis of late pachytene stage germ cells [42]. Directly scoring for the number of apoptotic corpses by DIC (differential interference contrast) microscopy revealed that apoptosis was increased in *mus-81* worms, further increased in *slx-4* worms, and that the highest level of apoptosis occurred in both *mus-81 lem-3* and *lem-3; slx-4* double mutant worms (Fig 2A). Apoptosis was reduced in *mus-81 lem-3; spo-11* triple mutants (Fig 2A). Careful examination of DAPI stained germ cell nuclei by fluorescence microscopy revealed that pyknotic cells, which have abnormally condensed nuclei, became apparent in mid/late pachytene in the *mus-81* single mutant and to a larger extent in the *mus-81 lem-3* and *lem-3; slx-4* double as well as the *mus-81 lem-3; spo-11 triple* mutant (Fig 2B). In *mus-81 lem-3; spo-11 triple* mutant, some pyknotic cells were already evident in the transition zone and early pachytene stages, where apoptotic cells are not apparent based on morphology under DIC microscopy [42, 43]. Given that mutation of *spo-11* does not fully bypass excessive apoptosis, some DNA lesions in *mus-81 lem-3* double mutants might be independent of the meiotic DSBs.

**Fig 3:**
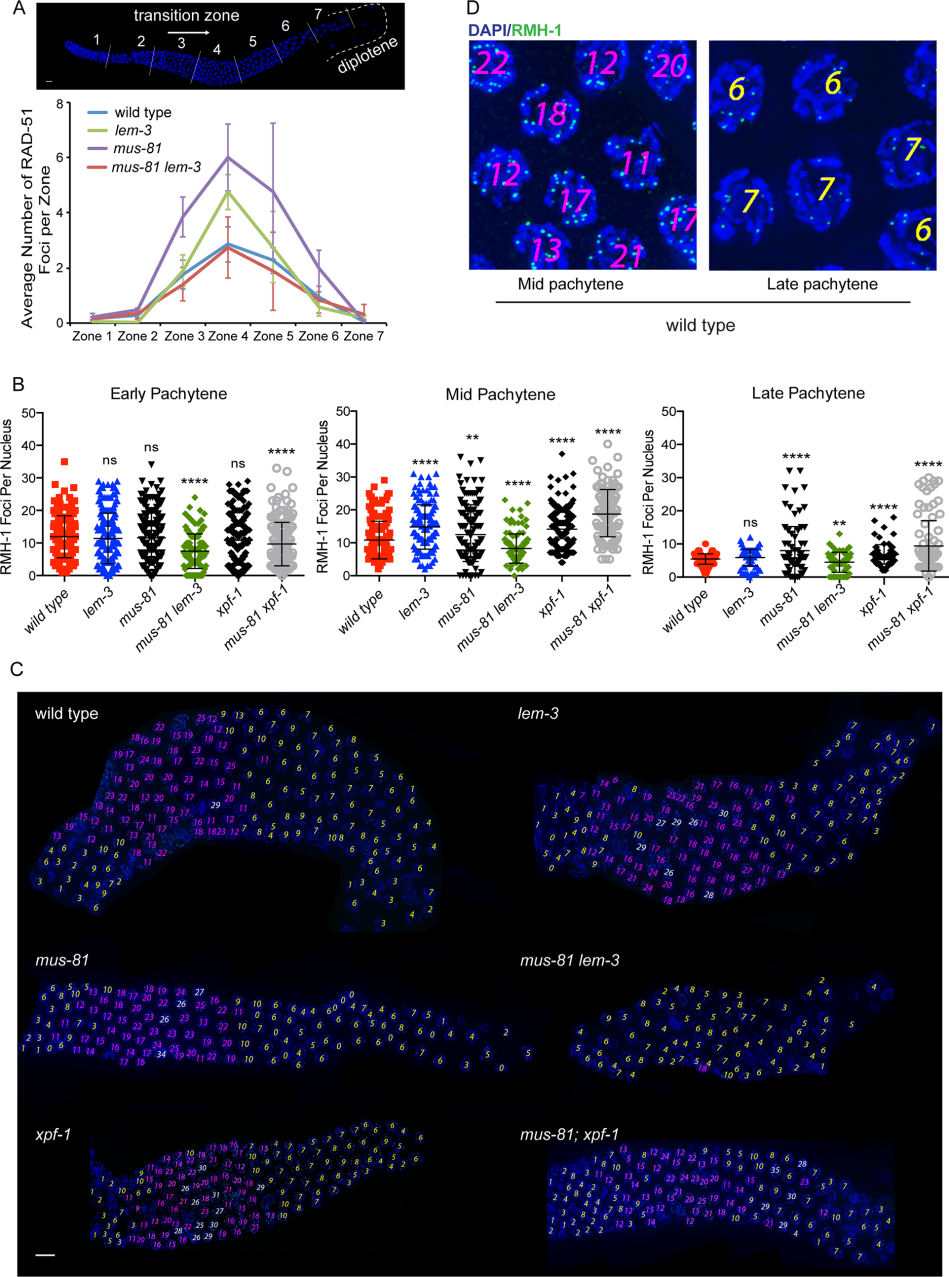
Comparison of RAD-51 and RMH-1 foci localization in wild type, *lem-3*, *mus-81* single mutants and *mus-81 lem-3* double mutant. (A) Quantification of RAD-51 profiles over the course of meiotic prophase. *C*. *elegans* gonads were divided into seven equal zones. We determined the number of RAD-51 foci in each zone. Quantifications were done based on three representative gonads for each genotype. Error bars are standard error of the mean. (B) Quantification of RMH-1::GFP foci in wild type, *lem-3*, *mus-81*, *xpf-1*,*mus-81 lem-3* and *mus-81; xpf-1* mutants in early Pachytene, mid Pachytene and late Pachytene stages. Quantifications were done for three gonads per genotype. Asterisks indicate statistical significance as determined by student T test. P Values below 0.05 were considered as significant, p < 0.05 is indicated with *, p < 0.01 with **, p < 0.005 with *** and p < 0.0001 with ****. (C) Representative images of gonads stained with DAPI. Yellow numbers represent those nuclei with RMH-1::GFP foci between 1 and 10, pink numbers represent nuclei with RMH-1::GFP counts between 11 and 25, and white numbers represent nuclei with RMH-1::GFP foci above 25. Scale bars: 5 μm. (D) Representative close-up images of mid and late Pachytene nuclei with different numbers of GFP::RMH-1 foci from a wild type gonad arm.

We next wished to analyse key recombination intermediates in *lem-3* single and *mus-81 lem-3* double mutant worms compared to wild type. During meiosis, an excess of meiotic DSBs are generated but most breaks are repaired without leading to a CO outcome and generally only one break for each chromosome pair matures into a CO-designated site in *C*. *elegans* [44]. RAD-51 foci mark early recombination intermediates engaged in strand invasion. RAD-51 foci accumulate in the transition zone where meiotic DSBs are initiated (Fig 3A, zone 3) and peak in early pachytene (Fig 3A, zone 4) [45]. We found that the number of RAD-51 foci was comparable to wild type in *mus-81 lem-3* double mutant worms, despite an increase of RAD-51 foci in *mus-81* and *lem-3* single mutants (Fig 3A). RMH-1 foci label both CO and NCO recombination intermediates and appear and disappear later than RAD-51, consistent with RMH-1 marking recombination intermediates after strand invasion [17]. We found that both *lem-3* and *mus-81* single mutants showed a wild type level of RMH-1 foci in early pachytene (with an average of 11 foci) while the number of foci seen in mid-pachytene in *lem-3* (on average 14.8 foci) and *mus-81* (12.9 foci) was higher than wild type (10.8 foci) (Fig 3B–3D), indicative of delayed recombination intermediate processing. Increased RMH-1 foci numbers were also observed in *xpf-1* single mutants and *mus-81; xpf-1* double mutants, in which, respectively, one or two redundant pathways needed to resolve meiotic HJ are compromised (Fig 3B–3D) [26–28], In contrast, the number of RHM-1 foci in *mus-81 lem-3* double mutants was significantly lower than wild type throughout the pachytene stage (Fig 3B and 3C). The number of recombination foci reflects the number of meiotic DSBs and the kinetics of their processing. Thus, our data suggest that LEM-3 and MUS-81 act in a similar step of DSB repair to ensure the proper maturation and/or turnover of meiotic recombination intermediates.

**Fig 4:**
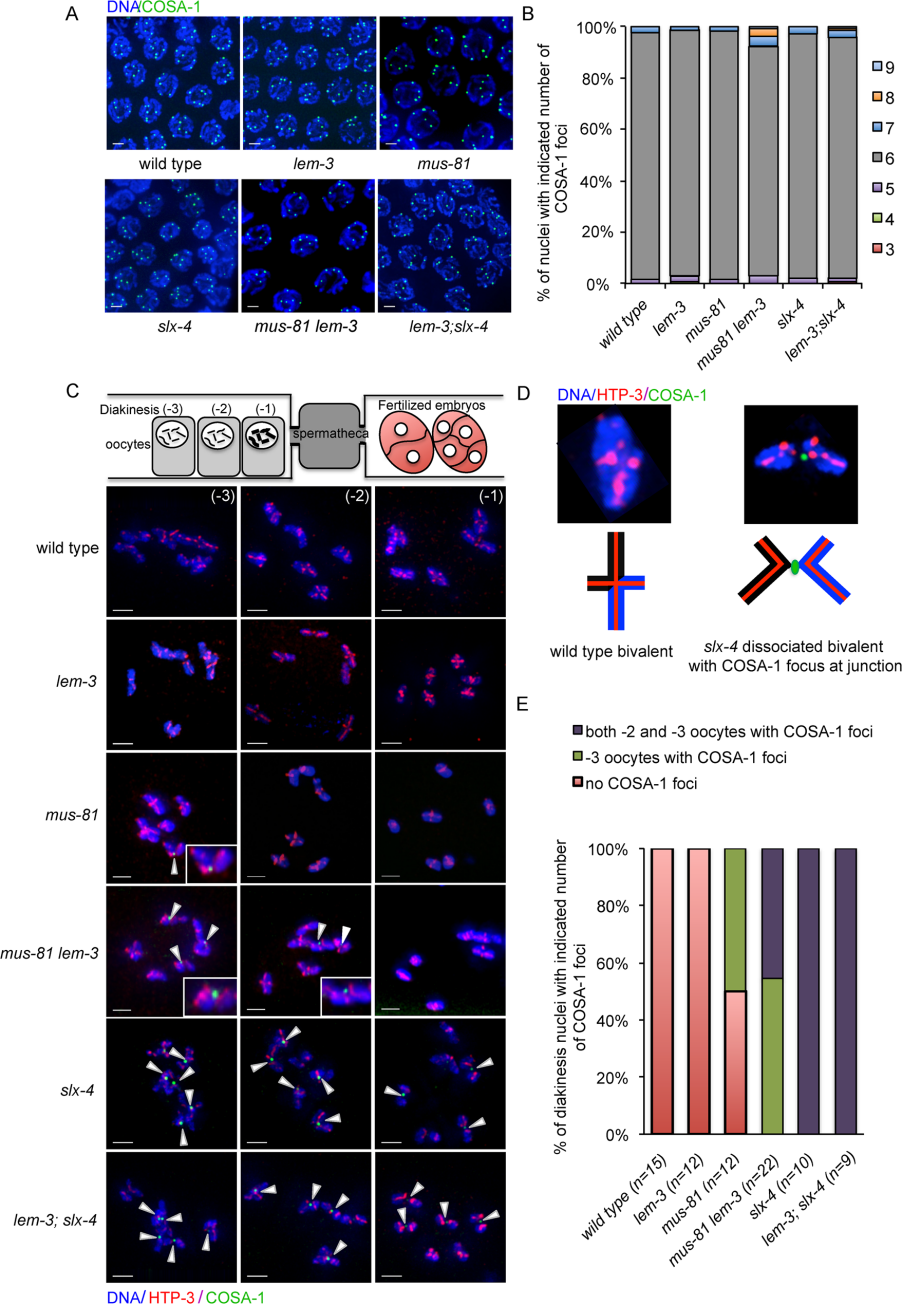
Delayed removal of COSA-1 foci in *mus-81 lem-3* double mutants. (A) Crossover designation is normal in *mus-81 lem-3* and *lem-3; slx-4* double mutants. DAPI staining of representative pachytene nuclei containing GFP::COSA-1 foci. Scale bars: 2 μm. (B) Quantification of nuclei with indicated number of COSA-1 foci. (C) Projections of representative nuclei from diakinesis oocytes of wild type, *lem-3*, *mus-81*, *slx-4*, *mus-81 lem-3* and *lem-3; slx-4* mutants stained with α-HTP-3, a component of the *C*. *elegans* axial element (red) and DAPI (blue). The persistent GFP::COSA-1 foci localized between two homologous pairs are highlighted by white arrowheads. (D) Scheme depicting normal bivalent from wild type and dissociated bivalent with COSA-1 focus at junction from *slx-4* mutant at the diakinesis stage. (E) Quantification of the diakinesisi nuclei with indicated number of COSA-1 foci. The sample size (n) indicates the number of germline examined for each genotype.

We also tested whether CO designation occurs normally using a strain expressing a functional GFP::COSA-1 fusion in *mus-81 lem-3* and *lem-3; slx-4* double mutant worms. COSA-1 foci mark CO designated sites in late pachytene [12]. As previously reported for wild type, *slx-4* and *mus-81* single mutants, also only 6 CO designated sites are apparent in the *lem-3* single and *lem-3; slx-4* double mutants in late pachytene (Fig 4A and 4B), indicating that CO designation is not perturbed. We note that while the majority of nuclei display 6 COSA-1 foci, a small number of nuclei with 7 COSA-1 foci (5/131 nuclei, p = 0.503 compared with 3/127 in wild type, not significant) and 8 COSA-1 foci (4/131 nuclei, p = 0.0477 compared with 0/127 in wild type) can be observed in *mus-81 lem-3* nuclei. In the wild type, COSA-1 protein develops as prominent foci at late pachytene and gradually dissociates from chromosome pairs in diplotene [12]. While COSA-1 foci fully disassemble in wild type and *lem-3* single mutant before the -3 oocyte (Fig 4C and 4E), COSA-1 foci could still be detected in 50% of -3 oocytes in *mus-81* single mutants *(n = 12)*. Strikingly, 45% of the -2 oocytes (n = 22) in *mus-81 lem-3* double mutants displayed COSA-1 foci, which eventually disappeared in -1 oocytes (Fig 4C and 4E). Persistent COSA-1 foci were also observed up to the -1 oocytes in *slx-4* single and *lem-3; slx-4* double mutants (Fig 4C and 4E). The COSA-1 foci localized to the chromosome linkages between dissociated bivalents, which could have been destined to become a CO (Fig 4D). Taken together, these data suggest that compromised recombination intermediate processing in *mus-81 lem-3* double mutant leads to a delay in the dismantling of COSA-1 foci.

**Fig 5:**
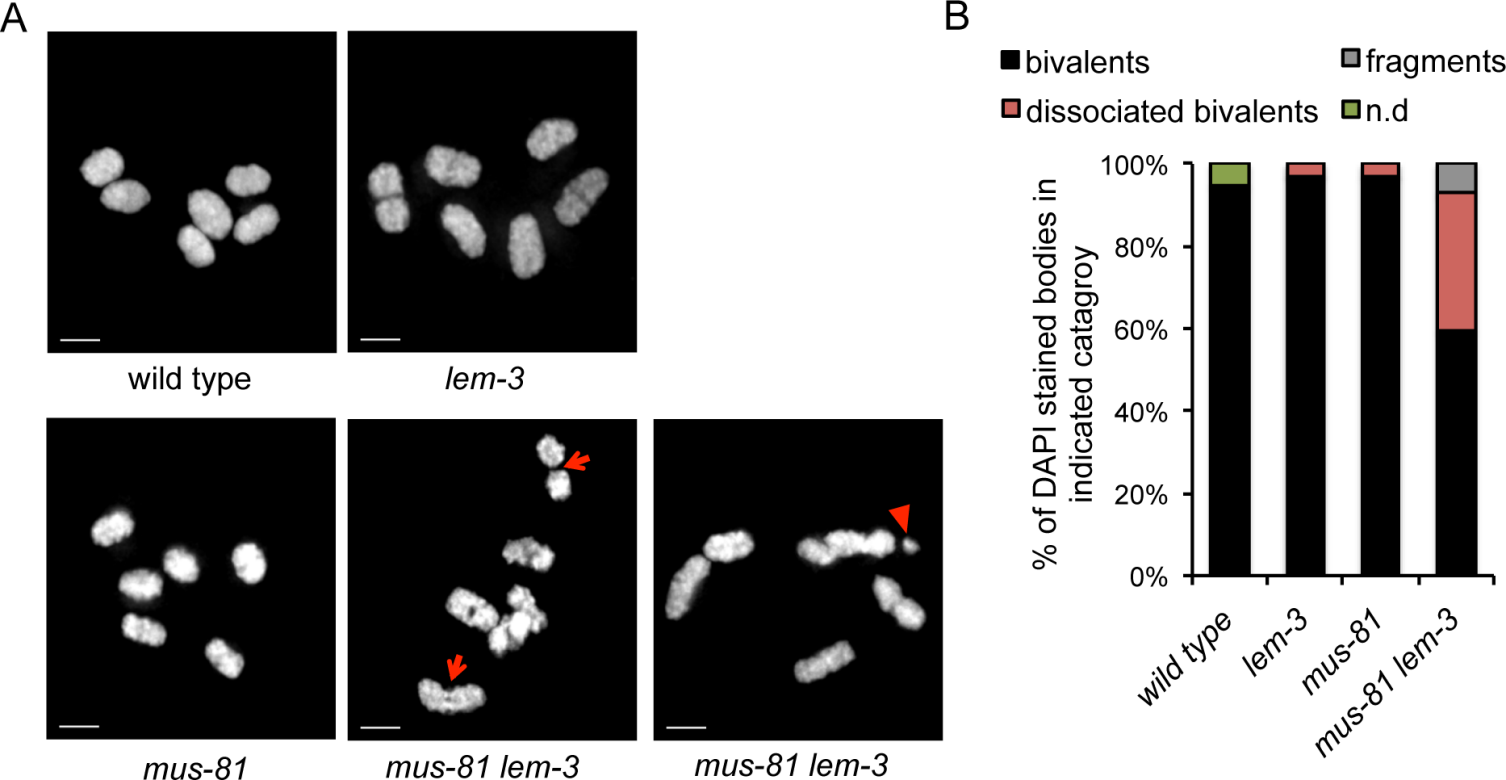
Depletion of LEM-3 and MUS-81 leads to formation of dissociated bivalents. (A) Images of DAPI-stained chromosomes in –1 oocytes at diakinesis in wild type, *lem-3*, *mus-81* and *mus-81 lem-3* mutants. Red arrows indicate dissociated bivalents. Chromosome fragment is highlighted with a red arrowhead. Scale bars: 2 μm. (B) Quantification of bivalents, ‘dissociated bivalents’ and fragments observed in indicated genotypes. Overlapping chromosomes that could not be assigned to the above categories were scored as “n/d”. Sample sizes of indicated genotype are as follows: wild type n = 40; *lem-3* n = 36; *mus-81* n = 36; *mus-81 lem-3* n = 42.

The morphology and number of diakinesis chromosomes can serve as readout for meiotic recombination defects. In diakinesis, homologous chromosome pairs restructure to form bivalents, which can be observed as 6 DAPI-stained bodies in wild type maturing oocytes [7]. Defects in meiotic recombination can result in a failure to stably connect homologous chromosomes, which becomes apparent as univalents at diakinesis (12 DAPI-stained bodies when physical linkages between all six homologue pairs fail to form). Our prior analysis of *slx-4*, as well as *mus-81; xpf-1* double mutants, revealed a distinct phenotype [26]. In contrast to *spo-11*, pairs of ‘univalents’ were found associated with each other, being linked by SPO-11 dependent chromatin bridges. We termed these structures as ´dissociated bivalents´. We interpreted these structures as chromosome pairs that engage in meiotic recombination but do not resolve recombination intermediates thus leading to the linkage of maternal and paternal chromosomes. As expected, wild type, *lem-3* and *mus-81* single mutants predominately had 6 bivalents (Fig 5). In contrast, *mus-81 lem-3* double mutant showed elevated numbers of dissociated bivalents (Fig 5, red arrow) and chromosome fragments (Fig 5, red arrowhead), which can be interpreted as unrepaired meiotic DSBs. The dissociated bivalents can also be detected in *slx-1 lem-3* mutants (S2 Fig). Importantly, the analysis of *slx-1 lem-3; spo-11* and *mus-81 lem-3; spo-11* triple mutants revealed 12 univalents (S2 Fig), indicating that the chromosome linkages we observed are *spo-11*-dependent and thus represent meiotic recombination intermediates.

### Elimination of *lem-3* does not lead to reduced CO recombination

The rate of CO recombination is reduced in *slx-1; xpf-1* and *mus-81; xpf-1* double mutants by approximately one third [26]. Given that *slx-1 lem-3*, *mus-81 lem-3* and *lem-3; slx-4* double mutants only sire dead embryos, we wanted to examine whether CO recombination is abolished in those double mutants. CO frequency and distribution can be investigated by meiotic recombination mapping [46]. We generated the *lem-3* single and double mutants with chromosome V being heterozygous for the Hawaiian and Bristol backgrounds. To determine the recombination frequency and distribution we employed five single nucleotide polymorphisms (snip-SNPs), which together cover 92% of chromosome V [26]. Given the lethality of various *lem-3* double mutants, embryos were used for recombination mapping to avoid biasing the analysis on the basis of viability. We found that CO recombination rates were comparable to wild type in *lem-3* single mutants (S3 Fig). Furthermore, when analysing the respective compound mutants, we found that *lem-3* did not lead to a decreased CO rate in conjunction with *mus-81* or *slx-4* single mutants (S3 Fig). Thus, despite the chromatin linkages that occur in various *lem-3* double mutants, CO recombination is not reduced.

### LEM-3 acts in conjunction with MUS-81 to prevent illegitimate recombination

We next investigated if LEM-3 was involved in inter-sister recombination repair pathway. Depletion of SYP-2, a component of the synaptonemal complex, abolishes the inter-homolog recombination and leads to the formation of 12 univalents, since breaks are likely repaired by using the sister chromatids as repair template (S4 Fig) [47]. If LEM-3 was able to promote inter-sister repair, *lem-3; syp-2* double mutants would be expected to have an increased number of DAPI-stained bodies at diakinesis; a phenotype indicative of chromosome fragmentation. However, we observed an average of 11.4 DAPI-stained bodies in *lem-3; syp-2* double mutants (S4 Fig). Furthermore, when we blocked the formation of the synaptonemal complex in *mus-81 lem-3* double mutants by *syp-2* RNAi, the average number of DAPI staining bodies did not increase (12.1, n = 19, p = 0.102 >0.05 compared to *syp-2* mutant). This result indicates that LEM-3 and MUS-81 are not involved in inter-sister recombination, or that a role in the resolution of inter-sister recombination intermediates is masked by a redundant pathway.

**Fig 6:**
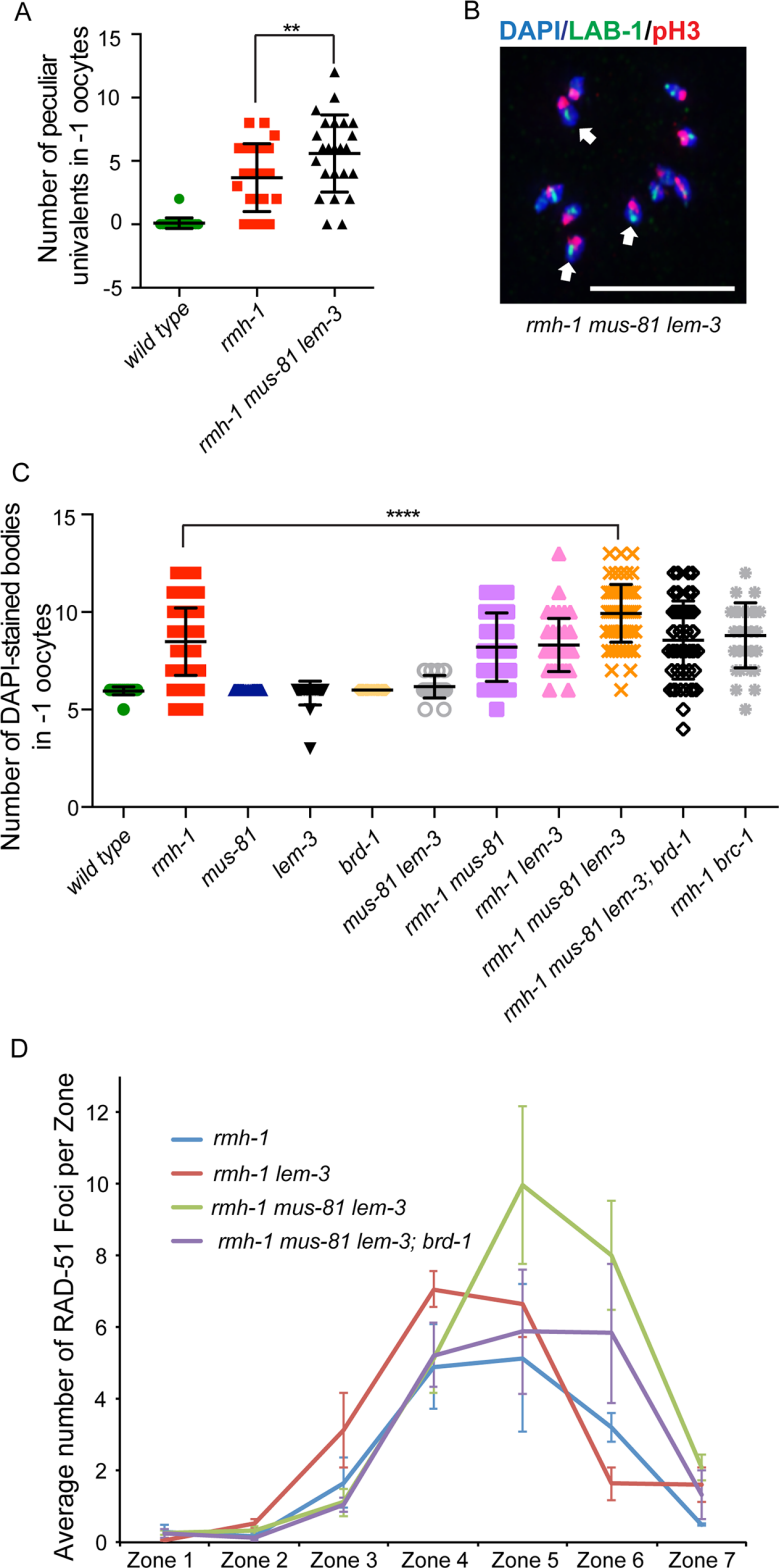
Univalent formation in *rmh-1 mus-81 lem-3* triple mutants can be reduced by depletion of *brd-1*. (A) Quantification of univalents in -1 oocytes of wild type, *rmh-1* and *rmh-1 mus-81 lem-3* mutants. The number of univalents in *rmh-1* and *rmh-1 mus-81 lem-3* mutants was counted. Asterisks indicate statistical significance as determined by Z-score. P Values below 0.05 were considered as significant, p < 0.05 is indicated with *, p < 0.01 with **, p < 0.005 with *** and p < 0.0001 with ****. (B) Representative image of a diakinesis nucleus of *rmh-1 mus-81 lem-3*. The majority of univalents classify as “peculiar univalents” (indicated by white arrow) stained by LAB-1 (green) and phsopho-histone H3 (pH3, red). Scale bars: 5 μm. (C) Quantification of DAPI-stained bodies in –1 oocytes at diakinesis in the indicated genotypes. The sample size (n) is indicated as follows: wild type (n = 23), *rmh-1* (n = 88), *mus-81* (n = 32), *lem-3* (n = 26), *brd-1* (n = 30), *mus-81 lem-3* (n = 23), *rmh-1 mus-81* (n = 46), *rmh-1 lem-3* (n = 40), *rmh-1 mus-81 lem-3* (n = 73), *rmh-1 mus-81 lem-3; brd-1* (n = 50). (D) Quantification of RAD-51 profiles over the course of meiotic prophase. *C*. *elegans* gonads were divided into seven equal zones and RAD-51 foci were counted in each nucleus of each zone. Quantifications were done based on three representative gonads per genotype. Error bars are standard error of the mean.

In *C*. *elegans*, COs trigger the restructuring of bivalents in CO distal and CO proximal domains revealed by the differential location of phospho-histone H3 (pH3) and the synapsis protein SYP-1 to the short arm, and the axial proteins LAB-1 (Long Arm of the Bivalent) and HTP-1/2 to the long arm of the 6 bivalents [48]. The lack of CO recombination as is the case in *spo-11* mutants, leads to the formation of 12 univalents without such bifurcation. In contrast, “peculiar univalents” display the same reciprocal localization as bivalents, suggesting that a CO site has been designated leading to enrichment of the above-mentioned markers to apposed chromosomal domains [17, 26, 36]. ‘Peculiar univalents’ were previously observed in *rmh-1*, *him-6 and xpf-1* single mutants [17, 26, 36]. We found an increased prevalence of univalents in *rmh-1 mus-81 lem-3* triple mutants compared to *rmh-1* single mutants, as revealed by an increased number of DAPI-stained bodies that display the features of “peculiar univalents” (Fig 6A and 6B, Please note, most quantification of univalents was done by DAPI staining, due to limited amounts of reagents to determine domain organization). Univalents were not detected in the *lem-3 or mus-81* single mutants as well as in *mus-81 lem-3* double mutants (Fig 6C) as evidenced by the 6 DAPI stained bodies we observed. In contrast, *rmh-1 mus-81 and rmh-1 lem-3* double mutants both showed an average of 8 DAPI stained bodies compared to the average of 10 observed in the *rmh-1 mus-81 lem-3* triple mutant (Fig 6C and S5 Fig, clearly discernible univalents are highlighted). Thus, the increased prevalence of univalents in *rmh-1 mus-81 lem-3* triple mutant might be the result of compromising several parallel recombination pathways.

We next set out to test if these univalents result from the mis-direction of recombination intermediates towards a NCO pathway such as inter-sister repair or SDSA. In *C*. *elegans*, the BRC-1 homologue of the mammalian BRCA1 recombination protein, which forms a heterodimer with BRD-1, has been proposed to be important for inter-sister repair in the germline [49]. Indeed, blocking inter-sister repair by introducing a *brd-1* mutation into *rmh-1 mus-81 lem-3* triple mutants resulted in a reduced number of univalents as revealed by an average of 8.5 DAPI stained bodies (Fig 6C and S5 Fig). The *rmh-1 mus-81 lem-3; brd-1* quadruple mutants also displayed a reduction of RAD-51 compared to *rmh-1 mus-81 lem-3* triple mutants, indicative of altered processing of meiotic recombination intermediates or reduced HR repair (Fig 6D). Altogether, these results indicate that LEM-3 might act in conjunction with MUS-81 and RMH-1 to process early recombination intermediate and the simultaneous absence of LEM-3, RMH-1 and MUS-81 could lead to illegitimate recombination intermediates impeding CO formation.

### LEM-3 promotes proper chromosome segregation during meiotic cell division

**Fig 7:**
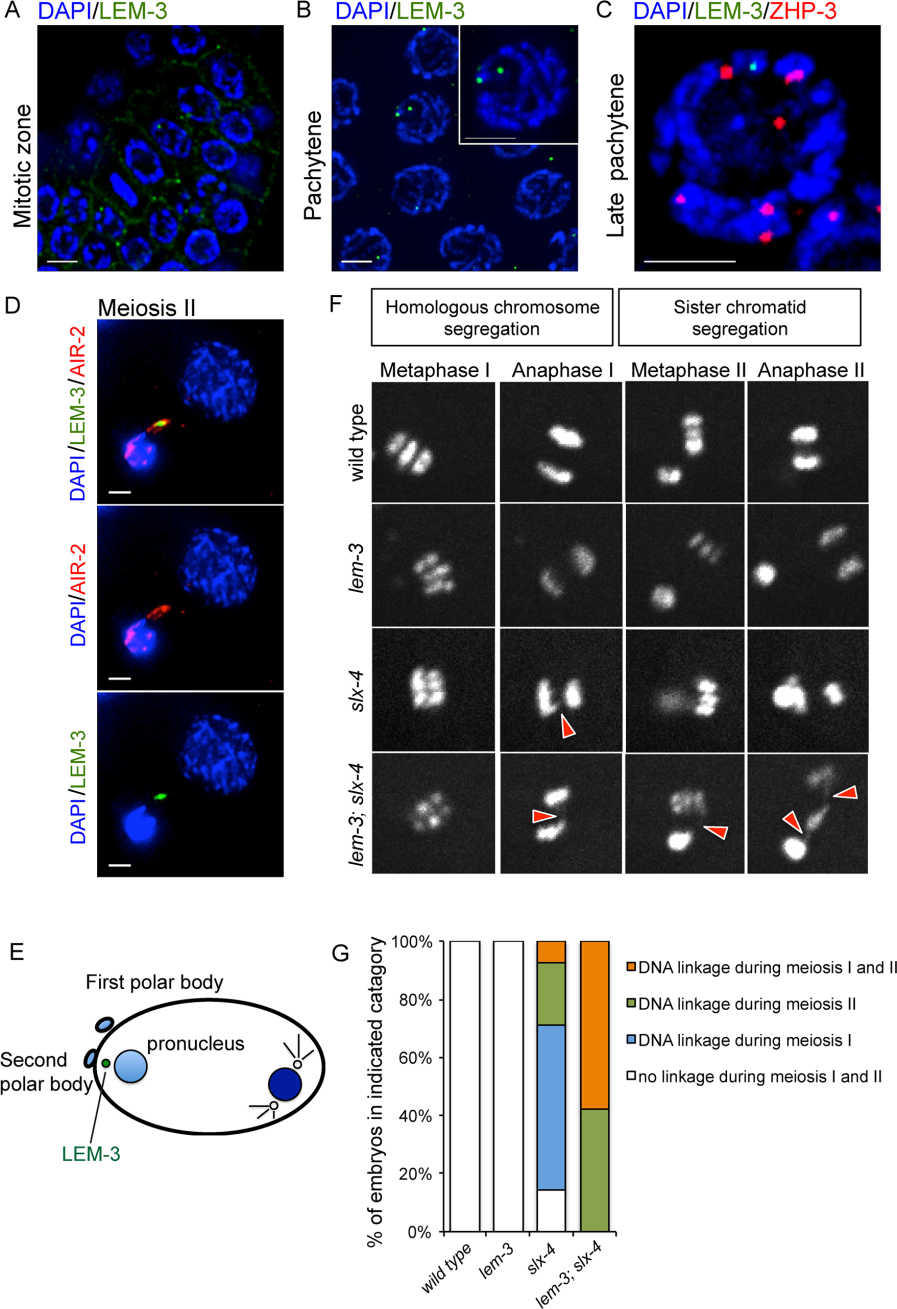
Localization of LEM-3 and its role in meiotic chromosome segregation. Localization of GFP::LEM-3 in mitotic zone (A) and pachytene stage (B). (C) LEM-3 foci (green) do not colocalize with crossover precursor marker ZHP-3 (red). (D) Colocalization of LEM-3 with AIR-2 between dividing nuclei during meiosis II. We note that the female pronucleus is already partially decondensed. (E) Schematic depiction of LEM-3 localization during meiotic division. (F) Representative images taken from time-lapse recordings of mCherry::Histone H2B expressing embryos during meiotic division. Red arrowheads indicate the chromatin linkages. (G) Quantification of DNA linkage formation during meiotic division in the indicated genotypes.

We next investigated the localization of LEM-3 using a strain expressing a GFP::LEM-3 fusion. Consistent with previous reports [37, 50], we found that LEM-3 localized as dots outside of the nucleus in the mitotic germ cells of wild type worms (Fig 7A). LEM-3 foci (typically no more than one per cell) were occasionally observed in pachytene, both in and outside of the nucleus (Fig 7B). These LEM-3 foci did not co-localize with the ZHP-3 marker that congresses into CO designated sites (Fig 7C) [51]. Interestingly, careful examination of cells undergoing meiotic divisions revealed that LEM-3 localized between dividing nuclei in meiosis II (Fig 7D and 7E). To analyse whether LEM-3 has a role in meiotic chromosome segregation, we performed live cell imaging of the first and second meiotic cell divisions by using an integrated Histone mCherry::H2B fusion. We reasoned that the chromosome segregation in meiosis I and II might be affected if a chromosome linkage remains present between two homologues, or sister chromatids, respectively. Chromosome segregation in the *lem-3* single mutant was similar to wild type (Fig 7F, S1 and S2 Movies). As we had previously reported for double mutants affecting both the SLX-1/MUS-81 and the HIM-6/XPF-1 pathway, chromosome linkages appeared during the first meiotic division in *slx-4* mutants (9/14 embryos, Fig 7F and 7G), consistent with an important role for SLX-4 in resolving inter-homolog recombination intermediates (Fig 7E and 7G, S3 Movie) [26] [48]. While the chromosome linkage could only be detected in 28.6% of *slx-4* mutant embryos (4/14) during the second meiotic division, all *lem-3; slx-4* double mutant embryos (19/19) showed extensive chromosome linkage formation in meiosis II (Fig 7F and 7G, S4 Movie). These data indicate that LEM-3 might have a role in processing recombination intermediates that persist into the second meiotic division.

## Discussion

In this study, we investigated the interplay between the *C*. *elegans* LEM-3/Ankle1 nuclease and nucleases previously implicated in meiotic CO resolution. We provide evidence for two roles of LEM-3 during meiosis. First, LEM-3 acts in conjunction with the MUS-81 and SLX-1-SLX-4 nucleases to process various recombination intermediates during meiotic prophase. Second, LEM-3 functions as a backup nuclease to deal with persistent DNA linkages during meiotic divisions.

The synthetic lethal interaction between LEM-3 and SLX-4 in *C*. *elegans* led us to investigate whether LEM-3 might act in parallel to the two identified redundant pathways for HJ resolution. Indeed, the lack of both LEM-3 and MUS-81 causes an increased number of dissociated bivalents (Fig 5), which represent unresolved recombination intermediates [26]. In addition, the profiles for major recombination markers are altered and CO maturation is delayed in *mus-81 lem-3* double mutants, as revealed by persistent COSA-1 foci at the CO designation sites in -2 oocytes (Fig 4C and 4E), suggesting that LEM-3 could be involved in processing of CO intermediates in the absence of CO resolvases such as MUS-81. In contrast, the *lem-3; xpf-1* double mutant showed no synthetic lethality, indicating that LEM-3 is able to process specific aberrant recombination intermediates that arise in *mus-81* mutants. However, the *lem-3* mutation in combination with *slx-4* or *mus-81* did not lead to a further reduction of CO frequency, or to an altered CO distribution (S3 Fig). Thus, synthetic lethality of the *mus-81 lem-3* mutant is not a result of decreased CO formation.

RMH-1 foci label both CO and NCO recombination intermediates. A previous study showed that RMH-1 does not colocalize with RAD-51 and appears and disappears later than RAD-51, suggesting that RMH-1 might act after RAD-51 removal and mark late recombination intermediates [17]. We found that the number of RMH-1 and RAD-51 foci was increased in *lem-3* and *mus-81* single mutants compared to wild type (Fig 3A and 3B), suggesting that both LEM-3 and MUS-81 play a role in the proper maturation/turnover of recombination intermediates. The increased number of RMH-1 and RAD-51 foci could be due to an increased number of processed DSBs, or due to a delay in DSB processing. Interestingly, the number of RAD-51 foci was comparable to wild type in the absence of both MUS-81 and LEM-3 (Fig 3A), indicating that the increased RAD-51 foci in *mus-81* single mutants is depended on the activity of LEM-3 and vice versa. In contrast, the number of RMH-1 foci was decreased in the *mus-81 lem-3* double mutant in early and mid pachytene compared to wild type (Fig 3B). These data suggest that MUS-81 and LEM-3 individually have a function in processing recombination intermediates and appear to act in conjunction after strand invasion but before the formation of RMH-1 foci. Irrespective, these alterations in the kinetics of RAD-51 or RMH-1 foci reflect the production and/or processing of early meiotic intermediates but do not overtly affect the number of CO designated sites. The number of COSA-1 foci that mark CO designated sites is roughly normal in the *mus-81 lem-3* double mutant (Fig 4A and 4B). Only in 3% of *mus-81 lem-3* mutant worms (4/131 nuclei) the number of COSA-1 foci is increased to 8, a finding which may indicate that the mechanisms leading the restriction to one CO per chromosome might be occasionally overwhelmed by unusual recombination intermediates occurring in this double mutant. Alternatively, this slightly increased number of COSA-1 foci could be linked to cells that become pyknotic and have abnormally condensed chromosomes and a higher number of smaller COSA-1 foci. Overall, our data suggest that LEM-3 and MUS-81 can process early recombination intermediates and contribute to the formation of recombination intermediates that recruit RMH-1.

How can we explain that *mus-81 lem-3* stains have dissociated bivalents but do not have a reduced rate of CO recombination? We interpreted these structures as chromosome pairs that engage in meiotic recombination but do not resolve recombination intermediates thus leading to the linkage of maternal and paternal chromosomes [26]. These structures were previously observed in *slx-4* single mutant worms, as well as in *mus-81; xpf-1* double mutants, and the CO rates were reduced as we expected [26, 48]. We favour two possible explanations for this discrepancy. DNA linkages might define CO intermediates that can still result in a NCO outcome. For instance, in the classical HJ resolution model, 4-way joint molecules that link maternal and paternal chromosomes can be resolved depending on the symmetry of the cleavage leading to CO or NCO outcome. Alternatively, it might as well be that chiasmata, the structures that hold maternal and paternal chromosomes together at the CO site, could be weakened in *mus-81 lem-3* double mutants, resulting in their dissociation. This dissociation could equally lead to a ‘dissociated bivalent’ phenotype. If such dissociation occurs, one does not expect reduced CO recombination. Both hypotheses are consistent with the delayed dissolution of COSA-1 foci.

How might the activity of LEM-3 be involved in processing meiotic recombination intermediates? It has been reported that LEM-3 and its human homologue ANKLE-1 are able to cleave supercoiled plasmid into relaxed circular (nicked) and linear DNA [37, 38]. In addition, LEM-3 can cleave a DNA substrate that is rich in secondary structures [37], indicating that LEM-3 might be a structure-specific endonuclease. Therefore, it is possible that LEM-3 can either process early recombination intermediates, such as D-loops, or else act at a late stage of meiotic recombination for HJ resolution to generate NCO products. It will be interesting to investigate the DNA substrate preference of LEM-3 in the future.

Chromosome segregation can be affected if unresolved recombination intermediates remain present during meiotic divisions. We previously observed that the extensive chromatin bridges generated by depleting both XPF-1 and MUS-81 pathways during the first meiotic division are eventually resolved in meiosis II, suggesting that backup activities function at or after anaphase I [26]. Here we found that LEM-3 localises between dividing nuclei during meiotic division (Fig 7D). In addition, depletion of LEM-3 leads to accumulation of chromosome linkages in the *slx-4* mutant, especially during meiosis II (Fig 7F), indicating that LEM-3 might have a role in proper chromosome segregation, by directly processing DNA linkages caused by unresolved recombination intermediates. A similar function has also been reported for the HJ resolvase GEN1/YEN1 nuclease in *S*. *cerevisiae* [52]. The budding yeast *yen1* mutant does not have obvious meiotic defects, whereas the *mus81 yen1* double mutant fails to segregate its chromosomes due to unresolved DNA joint-molecules during meiotic anaphase I and II [53]. Furthermore, the enzymatic activity of YEN1 is kept at a very low level in prophase but is highly induced at the onset of meiosis II, suggesting that it provides a safeguard activity that processes DNA linkages that escape the attention of MUS81 during meiotic divisions [53]. Mutation of the YEN1/GEN1 nuclease shows phenotypic variation in different organisms [21]. In *C*. *elegans*, no meiotic phenotypes were observed in the *gen-1* single mutant on its own or in combination with *him-6* or various nuclease mutants [54]. Our data suggest that LEM-3 might provide a failsafe system in *C*. *elegans* instead of GEN-1, to ensure that all recombination intermediates are resolved at the final stage of gamete formation. Consistent with this idea we recently provided evidence that LEM-3 might bind to chromatin bridges in the contractile ring in mitotic cells to process a large variety of DNA intermediates linked to recombination failure, DNA catenation, DNA decondensation failure, and to DNA underreplication [50]

In summary, we provide evidence for a role of LEM-3 in meiotic recombination intermediate processing in prophase I and in resolving persistent chromatin bridges during meiotic divisions. It will be interesting to see whether the mammalian LEM-3 orthologue Ankle1 has a role in meiosis.

## Materials and methods

### *C*. *elegans* strains and maintenance

Strains were grown at 20°C followed standard protocols [55]. N2 Bristol was used as the wild type. CB4856 Hawaii was used to generate strains for CO recombination frequency analysis. Strains used in this study are listed in S1 Table. The *cop859 [Plem-3*::*eGFP*::*STag*::*lem-3*::*3′UTRlem-3*] eGFP insertion was generated by Knudra (http://www.knudra.com/) following the procedures described by Dickinson et al [56]. Exact details are available upon request.

### Cytological procedures

Germline immunostaining was performed as described previously with slight modifications [35]. Primary and secondary antibodies were used at the indicated dilutions: rabbit anti-HTP-3 (1:500); guinea pig anti-ZHP-3 (1:250); rabbit anti-AIR-2 (1:200); rabbit anti-RAD-51 (1:1000); mouse anti-GFP (1:500); anti-rabbit Alexa488 (1:400) (Invitrogen), and anti-mouse Alexa488 (1:500) (Invitrogen) and anti-rabbit Alexa Fluor 568 (1:750) (Life technologies). For DAPI staining the final concentration used was 2 μg/mL.

### Recordings of meiotic divisions

Meiotic divisions were recorded by in utero embryo live imaging [57]. Worms were picked into a solution containing 1 mM levamisole to paralyze worms. Worms were mounted on 2% agar pads covered with a coverslip. Images were captured every 10 seconds using spinning-disk confocal microscopy.

### Image acquisition and analysis

Microscopy images acquired with a Delta Vision microscopy were deconvolved and analysed using softWoRx Suite and softWoRx Explorer software (AppliedPrecision, Issaquah, WA, USA). Images acquired with a spinning-disk confocal microscopy were analyzed by ImageJ software.

### RNA interference

RNAi was performed by feeding worms with bacteria containing plasmid that express double-stranded RNA for *syp-2* [58]. Worms were fed on NGM plates supplied with 100 mg/L ampicillin and 1mM IPTG. An empty L4440 plasmid was used as a control for RNAi experiment.

### Determining meiotic crossover frequency and distribution

Meiotic CO frequency and distribution were assayed essentially as described [26] with slight modifications. Five snip-SNPs on Chr. V that differ between N2 Bristol and CB4856 Hawaii were used to determine the crossover landscape in embryos. Single embryo was transferred into lysis buffer by mouth pipetting using a capillary and incubated at -80°C for at least 5 min to help crack the embryo before lysis.

## Supporting information

S1 Fig Meiotic chromosome axis formation is normal in *lem-3; slx-4* double mutants. Representative images of pachytene nuclei stained with an antibody recognizing the chromosome axis component HTP-3 (red) and DAPI (blue). Scale bars: 2 μm.
(TIF)

S2 Fig DNA linkages depend on *spo-11* induced meiotic double stranded breaks. White arrows indicate dissociated bivalents.
(TIF)

S3 Fig LEM-3 is dispensable for meiotic crossover formation. Analysis of crossover frequencies and distribution on chromosome V. The genetic map positions of the five SNPs, which together cover 92% of chromosome V, are indicated. n is the number of cross-progeny scored. The frequency of 2 COs, 1 CO or 0 CO per chromosome is indicated in absolute numbers and as percentage (in brackets).
(TIF)

S4 Fig Depletion of SYP-2 in *lem-3* mutants does not lead to chromosome fragmentation. (A) Representative images of DAPI-stained chromosomes from diakinesis oocytes of wild type, *lem-3*, *syp-2*, *lem-3; syp-2*, *mus-81; syp-2* RNAi and *mus-81 lem-3; syp-2* RNAi mutants. Scale bars: 2 μm. (B) Quantification of DAPI-stained bodies in diakinesis oocytes from indicated genotypes. Sample sizes of indicated genotype are as follows: wild type n = 32, *lem-3* n = 29, *syp-2* n = 29, *lem-3; syp-2* n = 34, *mus-81; syp-2* RNAi n = 14 and *mus-81 syp-2* n = 19. n.s.: not significant, p = 0.053.
(TIF)

S5 Fig Representative images of DAPI-stained diakinesis chromosomes of indicated genotypes. Univalents are indicated by red arrowheads. Scale bar: 5 μm.
(TIF)

S1 Movie Wild type mCherry::H2B, meiosis I and II. Video shows an embryo expressing mCherry-Histone H2B progressing throughout the first and second meiotic divisions. Images were acquired every 10 sec with a spinning disk confocal microscope and processed with ImageJ software.
(MOV)

S2 Movie *lem-3*; mCherry::H2B, meiosis I and II. Images were acquired and analyzed as Movie S1.
(MOV)

S3 Movie *slx-4*; mCherry::H2B, meiosis I and II. Images were acquired and analyzed as S1 Movie.
(MOV)

S4 Movie *lem-3; slx-4* mCherry::H2B, meiosis I and II. Images were acquired and analyzed as S1 Movie.
(MOV)

S1 Table List of strains used in this study. (DOCX)

## References

[1] Kohl KP, Sekelsky J. Meiotic and mitotic recombination in meiosis. Genetics. 2013;194(2):327–34. Epub 2013/06/05. doi: 10.1534/genetics.113.150581 ; PubMed Central PMCID: PMC3664844. 23733849 10.1534/genetics.113.150581 PMC3664844

[2] Keeney S. Spo11 and the formation of DNA double-strand breaks in meiosis. Genome Dyn Stab. 2008;2:81–123. Epub 2008/01/01. doi: 10.1007/7050_2007_026 ; PubMed Central PMCID: PMC3172816. 21927624 10.1007/7050_2007_026 PMC3172816

[3] Buhler C, Borde V, Lichten M. Mapping meiotic single-strand DNA reveals a new landscape of DNA double-strand breaks in *Saccharomyces cerevisiae*. PLoS Biol. 2007;5(12):e324 Epub 2007/12/14. doi: 10.1371/journal.pbio.0060104 ; PubMed Central PMCID: PMC2121111. 18076285 10.1371/journal.pbio.0050324 PMC2121111

[4] Moens PB, Kolas NK, Tarsounas M, Marcon E, Cohen PE, Spyropoulos B. The time course and chromosomal localization of recombination-related proteins at meiosis in the mouse are compatible with models that can resolve the early DNA-DNA interactions without reciprocal recombination. J Cell Sci. 2002;115(Pt 8):1611–22. Epub 2002/04/16. . 11950880 10.1242/jcs.115.8.1611

[5] Rosu S, Libuda DE, Villeneuve AM. Robust crossover assurance and regulated interhomolog access maintain meiotic crossover number. Science. 2011;334(6060):1286–9. Epub 2011/12/07. doi: 10.1126/science.1212424 ; PubMed Central PMCID: PMC3360972. 22144627 10.1126/science.1212424 PMC3360972

[6] Franklin AE, McElver J, Sunjevaric I, Rothstein R, Bowen B, Cande WZ. Three-dimensional microscopy of the Rad51 recombination protein during meiotic prophase. Plant cell. 1999;11(5):809–24. Epub 1999/05/20. ; PubMed Central PMCID: PMC144225. 10330467 10.1105/tpc.11.5.809 PMC144225

[7] Lui DY, Colaiacovo MP. Meiotic development in *Caenorhabditis elegans*. Adv Exp Med Biol. 2013;757:133–70. Epub 2012/08/09. doi: 10.1007/978-1-4614-4015-4_6 ; PubMed Central PMCID: PMC3764601. 22872477 10.1007/978-1-4614-4015-4_6 PMC3764601

[8] Woglar A, Villeneuve AM. Dynamic Architecture of DNA Repair Complexes and the Synaptonemal Complex at Sites of Meiotic Recombination. Cell. 2018;173:1–14. 29754818 10.1016/j.cell.2018.03.066 PMC6003859

[9] Yu Z, Kim Y, Dernburg AF. Meiotic recombination and the crossover assurance checkpoint in *Caenorhabditis elegans*. Semin Cell Dev Biol. 2016;54:106–16. Epub 2016/03/26. doi: 10.1016/j.semcdb.2016.03.014 ; PubMed Central PMCID: PMC5082714. 27013114 10.1016/j.semcdb.2016.03.014 PMC5082714

[10] Stamper EL, Rodenbusch SE, Rosu S, Ahringer J, Villeneuve AM, Dernburg AF. Identification of DSB-1, a protein required for initiation of meiotic recombination in *Caenorhabditis elegans*, illuminates a crossover assurance checkpoint. PLoS Genet. 2013;9(8):e1003679 Epub 2013/08/31. doi: 10.1371/journal.pgen.1003679 ; PubMed Central PMCID: PMC3749324. 23990794 10.1371/journal.pgen.1003679 PMC3749324

[11] Rosu S, Zawadzki KA, Stamper EL, Libuda DE, Reese AL, Dernburg AF, et al The *C*. *elegans* DSB-2 protein reveals a regulatory network that controls competence for meiotic DSB formation and promotes crossover assurance. PLoS Genet. 2013;9(8):e1003674 Epub 2013/08/21. doi: 10.1371/journal.pgen.1003674 ; PubMed Central PMCID: PMC3738457. 23950729 10.1371/journal.pgen.1003674 PMC3738457

[12] Yokoo R, Zawadzki KA, Nabeshima K, Drake M, Arur S, Villeneuve AM. COSA-1 reveals robust homeostasis and separable licensing and reinforcement steps governing meiotic crossovers. Cell. 2012;149(1):75–87. Epub 2012/04/03. doi: 10.1016/j.cell.2012.01.052 ; PubMed Central PMCID: PMC3339199. 22464324 10.1016/j.cell.2012.01.052 PMC3339199

[13] Holloway JK, Sun X, Yokoo R, Villeneuve AM, Cohen PE. Mammalian CNTD1 is critical for meiotic crossover maturation and deselection of excess precrossover sites. J Cell Biol. 2014;205(5):633–41. Epub 2014/06/04. doi: 10.1083/jcb.201401122 ; PubMed Central PMCID: PMC4050721. 24891606 10.1083/jcb.201401122 PMC4050721

[14] Reynolds A, Qiao H, Yang Y, Chen JK, Jackson N, Biswas K, et al RNF212 is a dosage-sensitive regulator of crossing-over during mammalian meiosis. Nature Genet. 2013;45(3):269–78. Epub 2013/02/12. doi: 10.1038/ng.2541 ; PubMed Central PMCID: PMC4245152. 23396135 10.1038/ng.2541 PMC4245152

[15] Qiao H, Prasada Rao HB, Yang Y, Fong JH, Cloutier JM, Deacon DC, et al Antagonistic roles of ubiquitin ligase HEI10 and SUMO ligase RNF212 regulate meiotic recombination. Nature Genet. 2014;46(2):194–9. Epub 2014/01/07. doi: 10.1038/ng.2858 ; PubMed Central PMCID: PMC4356240. 24390283 10.1038/ng.2858 PMC4356240

[16] Zalevsky J, MacQueen AJ, Duffy JB, Kemphues KJ, Villeneuve AM. Crossing over during Caenorhabditis elegans meiosis requires a conserved MutS-based pathway that is partially dispensable in budding yeast. Genetics. 1999;153(3):1271–83. Epub 1999/11/05. ; PubMed Central PMCID: PMC1460811. 10545458 10.1093/genetics/153.3.1271 PMC1460811

[17] Jagut M, Hamminger P, Woglar A, Millonigg S, Paulin L, Mikl M, et al Separable roles for a *Caenorhabditis elegans* RMI1 homolog in promoting and antagonizing meiotic crossovers ensure faithful chromosome inheritance. PLoS Biol. 2016;14(3):e1002412 Epub 2016/03/25. doi: 10.1371/journal.pbio.1002412 ; PubMed Central PMCID: PMC4807110. 27011106 10.1371/journal.pbio.1002412 PMC4807110

[18] Shinagawa H, Iwasaki H. Processing the Holliday junction in homologous recombination. Trends Biochem Sci. 1996;21(3):107–11. Epub 1996/03/01. . 8882584

[19] Schwacha A, Kleckner N. Identification of double Holliday junctions as intermediates in meiotic recombination. Cell. 1995;83(5):783–91. Epub 1995/12/01. . 8521495 10.1016/0092-8674(95)90191-4

[20] Heyer WD. Recombination: Holliday junction resolution and crossover formation. Curr Biol: CB. 2004;14(2):R56–8. Epub 2004/01/24. . 14738748 10.1016/j.cub.2003.12.043

[21] Wyatt HD, West SC. Holliday junction resolvases. Cold Spring Harb Perspect Biol. 2014;6(9):a023192 Epub 2014/09/04. doi: 10.1101/cshperspect.a023192 ; PubMed Central PMCID: PMC4142969. 25183833 10.1101/cshperspect.a023192 PMC4142969

[22] Osman F, Dixon J, Doe CL, Whitby MC. Generating crossovers by resolution of nicked Holliday junctions: a role for Mus81-Eme1 in meiosis. Mol cell. 2003;12(3):761–74. Epub 2003/10/07. . 14527420 10.1016/s1097-2765(03)00343-5

[23] Zakharyevich K, Tang S, Ma Y, Hunter N. Delineation of joint molecule resolution pathways in meiosis identifies a crossover-specific resolvase. Cell. 2012;149(2):334–47. Epub 2012/04/17. doi: 10.1016/j.cell.2012.03.023 ; PubMed Central PMCID: PMC3377385. 22500800 10.1016/j.cell.2012.03.023 PMC3377385

[24] Zakharyevich K, Ma Y, Tang S, Hwang PY, Boiteux S, Hunter N. Temporally and biochemically distinct activities of Exo1 during meiosis: double-strand break resection and resolution of double Holliday junctions. Mol cell. 2010;40(6):1001–15. Epub 2010/12/22. doi: 10.1016/j.molcel.2010.11.032 ; PubMed Central PMCID: PMC3061447. 21172664 10.1016/j.molcel.2010.11.032 PMC3061447

[25] Holloway JK, Booth J, Edelmann W, McGowan CH, Cohen PE. MUS81 generates a subset of MLH1-MLH3-independent crossovers in mammalian meiosis. PLoS Genet. 2008;4(9):e1000186 Epub 2008/09/13. doi: 10.1371/journal.pgen.1000186 ; PubMed Central PMCID: PMC2525838. 18787696 10.1371/journal.pgen.1000186 PMC2525838

[26] Agostinho A, Meier B, Sonneville R, Jagut M, Woglar A, Blow J, et al Combinatorial regulation of meiotic holliday junction resolution in C. elegans by HIM-6 (BLM) helicase, SLX-4, and the SLX-1, MUS-81 and XPF-1 nucleases. PLoS Genet. 2013;9(7):e1003591 Epub 2013/08/01. doi: 10.1371/journal.pgen.1003591 ; PubMed Central PMCID: PMC3715425. 23901331 10.1371/journal.pgen.1003591 PMC3715425

[27] O'Neil NJ, Martin JS, Youds JL, Ward JD, Petalcorin MI, Rose AM, et al Joint molecule resolution requires the redundant activities of MUS-81 and XPF-1 during *Caenorhabditis elegans* meiosis. PLoS Genet. 2013;9(7):e1003582 Epub 2013/07/23. doi: 10.1371/journal.pgen.1003582 ; PubMed Central PMCID: PMC3715453. 23874209 10.1371/journal.pgen.1003582 PMC3715453

[28] Saito TT, Lui DY, Kim HM, Meyer K, Colaiacovo MP. Interplay between structure-specific endonucleases for crossover control during *Caenorhabditis elegans* meiosis. PLoS Genet. 2013;9(7):e1003586 Epub 2013/07/23. doi: 10.1371/journal.pgen.1003586 ; PubMed Central PMCID: PMC3715419. 23874210 10.1371/journal.pgen.1003586 PMC3715419

[29] Wyatt HD, Sarbajna S, Matos J, West SC. Coordinated actions of SLX1-SLX4 and MUS81-EME1 for Holliday junction resolution in human cells. Mol cell. 2013;52(2):234–47. Epub 2013/10/01. doi: 10.1016/j.molcel.2013.08.035 . 24076221 10.1016/j.molcel.2013.08.035

[30] Martinez-Perez E, Colaiacovo MP. Distribution of meiotic recombination events: talking to your neighbors. Curr Opin Genet Dev. 2009;19(2):105–12. Epub 2009/03/31. doi: 10.1016/j.gde.2009.02.005 ; PubMed Central PMCID: PMC2729281. 19328674 10.1016/j.gde.2009.02.005 PMC2729281

[31] Hunter N, Kleckner N. The single-end invasion: an asymmetric intermediate at the double-strand break to double-Holliday junction transition of meiotic recombination. Cell. 2001;106(1):59–70. Epub 2001/07/20. . 11461702 10.1016/s0092-8674(01)00430-5

[32] Bishop DK, Zickler D. Early decision; meiotic crossover interference prior to stable strand exchange and synapsis. Cell. 2004;117(1):9–15. Epub 2004/04/07. . 15066278 10.1016/s0092-8674(04)00297-1

[33] Ira G, Malkova A, Liberi G, Foiani M, Haber JE. Srs2 and Sgs1-Top3 suppress crossovers during double-strand break repair in yeast. Cell. 2003;115(4):401–11. Epub 2003/11/19. ; PubMed Central PMCID: PMC4493758. 14622595 10.1016/s0092-8674(03)00886-9 PMC4493758

[34] Youds JL, Mets DG, McIlwraith MJ, Martin JS, Ward JD, NJ ON, et al RTEL-1 enforces meiotic crossover interference and homeostasis. Science. 2010;327(5970):1254–8. Epub 2010/03/06. doi: 10.1126/science.1183112 ; PubMed Central PMCID: PMC4770885. 20203049 10.1126/science.1183112 PMC4770885

[35] Hong Y, Sonneville R, Agostinho A, Meier B, Wang B, Blow JJ, et al The SMC-5/6 complex and the HIM-6 (BLM) helicase synergistically promote meiotic recombination intermediate processing and chromosome maturation during *Caenorhabditis elegans* meiosis. PLoS Genet. 2016;12(3):e1005872 Epub 2016/03/25. doi: 10.1371/journal.pgen.1005872 ; PubMed Central PMCID: PMC4807058. 27010650 10.1371/journal.pgen.1005872 PMC4807058

[36] Schvarzstein M, Pattabiraman D, Libuda DE, Ramadugu A, Tam A, Martinez-Perez E, et al DNA helicase HIM-6/BLM both promotes MutSgamma-dependent crossovers and antagonizes MutSgamma-independent interhomolog associations during caenorhabditis elegans meiosis. Genetics. 2014;198(1):193–207. Epub 2014/07/24. doi: 10.1534/genetics.114.161513 ; PubMed Central PMCID: PMC4174932. 25053665 10.1534/genetics.114.161513 PMC4174932

[37] Dittrich CM, Kratz K, Sendoel A, Gruenbaum Y, Jiricny J, Hengartner MO. LEM-3—A LEM domain containing nuclease involved in the DNA damage response in *C*. *elegans*. PloS one. 2012;7(2):e24555 Epub 2012/03/03. doi: 10.1371/journal.pone.0024555 ; PubMed Central PMCID: PMC3285610. 22383942 10.1371/journal.pone.0024555 PMC3285610

[38] Brachner A, Braun J, Ghodgaonkar M, Castor D, Zlopasa L, Ehrlich V, et al The endonuclease Ankle1 requires its LEM and GIY-YIG motifs for DNA cleavage in vivo. J Cell Sci. 2012;125(Pt 4):1048–57. Epub 2012/03/09. doi: 10.1242/jcs.098392 ; PubMed Central PMCID: PMC4335191. 22399800 10.1242/jcs.098392 PMC4335191

[39] Braun J, Meixner A, Brachner A, Foisner R. The GIY-YIG Type Endonuclease Ankyrin Repeat and LEM Domain-Containing Protein 1 (ANKLE1) Is Dispensable for Mouse Hematopoiesis. PLoS One. 2016;11(3):e0152278 Epub 2016/03/25. doi: 10.1371/journal.pone.0152278 ; PubMed Central PMCID: PMC4807109. 27010503 10.1371/journal.pone.0152278 PMC4807109

[40] Severson AF, Ling L, van Zuylen V, Meyer BJ. The axial element protein HTP-3 promotes cohesin loading and meiotic axis assembly in *C*. *elegans* to implement the meiotic program of chromosome segregation. Genes Dev. 2009;23(15):1763–78. Epub 2009/07/04. doi: 10.1101/gad.1808809 ; PubMed Central PMCID: PMC2720254. 19574299 10.1101/gad.1808809 PMC2720254

[41] Kim Y, Rosenberg SC, Kugel CL, Kostow N, Rog O, Davydov V, et al The chromosome axis controls meiotic events through a hierarchical assembly of HORMA domain proteins. Dev cell. 2014;31(4):487–502. Epub 2014/12/03. doi: 10.1016/j.devcel.2014.09.013 ; PubMed Central PMCID: PMC4254552. 25446517 10.1016/j.devcel.2014.09.013 PMC4254552

[42] Gartner A, Milstein S, Ahmed S, Hodgkin J, Hengartner MO. A conserved checkpoint pathway mediates DNA damage—induced apoptosis and cell cycle arrest in *C*. *elegans*. Mol cell. 2000;5(3):435–43. Epub 2000/07/06. . 10882129 10.1016/s1097-2765(00)80438-4

[43] Schumacher B, Hanazawa M, Lee MH, Nayak S, Volkmann K, Hofmann ER, et al Translational repression of *C*. *elegans* p53 by GLD-1 regulates DNA damage-induced apoptosis. Cell. 2005;120(3):357–68. Epub 2005/02/15. doi: 10.1016/j.cell.2004.12.009 . 15707894 10.1016/j.cell.2004.12.009

[44] Hillers KJ, Villeneuve AM. Chromosome-wide control of meiotic crossing over in C. elegans. Curr Biol: CB. 2003;13(18):1641–7. Epub 2003/09/19. . 13678597 10.1016/j.cub.2003.08.026

[45] Alpi A, Pasierbek P, Gartner A, Loidl J. Genetic and cytological characterization of the recombination protein RAD-51 in *Caenorhabditis elegans*. Chromosoma. 2003;112(1):6–16. Epub 2003/04/10. doi: 10.1007/s00412-003-0237-5 . 12684824 10.1007/s00412-003-0237-5

[46] Hillers KJ, Villeneuve AM. Analysis of meiotic recombination in *Caenorhabditis elegans*. Methods Mol Biol. 2009;557:77–97. Epub 2009/10/06. doi: 10.1007/978-1-59745-527-5_7 . 19799178 10.1007/978-1-59745-527-5_7

[47] Colaiacovo MP, MacQueen AJ, Martinez-Perez E, McDonald K, Adamo A, La Volpe A, et al Synaptonemal complex assembly in *C*. *elegans* is dispensable for loading strand-exchange proteins but critical for proper completion of recombination. Dev Cell. 2003;5(3):463–74. doi: 10.1016/S1534-5807(03)00232-6 PubMed PMID: ISI:000185309600014. 12967565 10.1016/s1534-5807(03)00232-6

[48] Saito TT, Youds JL, Boulton SJ, Colaiacovo MP. *Caenorhabditis elegans* HIM-18/SLX-4 interacts with SLX-1 and XPF-1 and maintains genomic integrity in the germline by processing recombination intermediates. PLoS Genet. 2009;5(11):e1000735 Epub 2009/11/26. doi: 10.1371/journal.pgen.1000735 ; PubMed Central PMCID: PMC2770170. 19936019 10.1371/journal.pgen.1000735 PMC2770170

[49] Adamo A, Montemauri P, Silva N, Ward JD, Boulton SJ, La Volpe A. BRC-1 acts in the inter-sister pathway of meiotic double-strand break repair. EMBO Rep. 2008;9(3):287–92. Epub 2008/01/26. doi: 10.1038/sj.embor.7401167 ; PubMed Central PMCID: PMC2267377. 18219312 10.1038/sj.embor.7401167 PMC2267377

[50] Hong Y, Sonneville R, Wang B, Scheidt V, Meier B, Woglar A, et al LEM-3 is a midbody-tethered DNA nuclease that resolves chromatin bridges during late mitosis. Nature Commun. 2018;9(1):728 Epub 2018/02/22. doi: 10.1038/s41467-018-03135-w . 29463814 10.1038/s41467-018-03135-w PMC5820297

[51] Bhalla N, Wynne DJ, Jantsch V, Dernburg AF. ZHP-3 acts at crossovers to couple meiotic recombination with synaptonemal complex disassembly and bivalent formation in *C*. *elegans*. PLoS Genet. 2008;4(10):e1000235 Epub 2008/10/25. doi: 10.1371/journal.pgen.1000235 ; PubMed Central PMCID: PMC2567099. 18949042 10.1371/journal.pgen.1000235 PMC2567099

[52] Ip SC, Rass U, Blanco MG, Flynn HR, Skehel JM, West SC. Identification of Holliday junction resolvases from humans and yeast. Nature. 2008;456(7220):357–61. Epub 2008/11/21. doi: 10.1038/nature07470 . 19020614 10.1038/nature07470

[53] Matos J, Blanco MG, Maslen S, Skehel JM, West SC. Regulatory control of the resolution of DNA recombination intermediates during meiosis and mitosis. Cell. 2011;147(1):158–72. Epub 2011/10/04. doi: 10.1016/j.cell.2011.08.032 ; PubMed Central PMCID: PMC3560330. 21962513 10.1016/j.cell.2011.08.032 PMC3560330

[54] Bailly AP, Freeman A, Hall J, Declais AC, Alpi A, Lilley DM, et al The *Caenorhabditis elegans* homolog of Gen1/Yen1 resolvases links DNA damage signaling to DNA double-strand break repair. PLoS Genet. 2010;6(7):e1001025 Epub 2010/07/28. doi: 10.1371/journal.pgen.1001025 ; PubMed Central PMCID: PMC2908289. 20661466 10.1371/journal.pgen.1001025 PMC2908289

[55] Brenner S. The genetics of *Caenorhabditis elegans*. Genetics. 1974;77(1):71–94. Epub 1974/05/01. ; PubMed Central PMCID: PMC1213120. 4366476 10.1093/genetics/77.1.71 PMC1213120

[56] Dickinson DJ, Pani AM, Heppert JK, Higgins CD, Goldstein B. Streamlined Genome Engineering with a Self-Excising Drug Selection Cassette. Genetics. 2015;200(4):1035–49. Epub 2015/06/06. doi: 10.1534/genetics.115.178335 ; PubMed Central PMCID: PMC4574250. 26044593 10.1534/genetics.115.178335 PMC4574250

[57] Pelisch F, Tammsalu T, Wang B, Jaffray EG, Gartner A, Hay RT. A SUMO-dependent protein network regulates chromosome congression during oocyte meiosis. Mol cell. 2017;65(1):66–77. Epub 2016/12/13. doi: 10.1016/j.molcel.2016.11.001 ; PubMed Central PMCID: PMC5222697. 27939944 10.1016/j.molcel.2016.11.001 PMC5222697

[58] Timmons L, Fire A. Specific interference by ingested dsRNA. Nature. 1998;395(6705):854 Epub 1998/11/06. doi: 10.1038/27579 . 9804418 10.1038/27579

