# Supplementary figures and images for: The conserved LEM-3/Ankle1 nuclease is involved in the combinatorial regulation of meiotic recombination repair and chromosome segregation in Caenorhabditis elegans
Source: PLoS Genet. 2018 Jun 7;14(6):e1007453. doi: 10.1371/journal.pgen.1007453 (PMC6007928; doi:10.1371/journal.pgen.1007453)

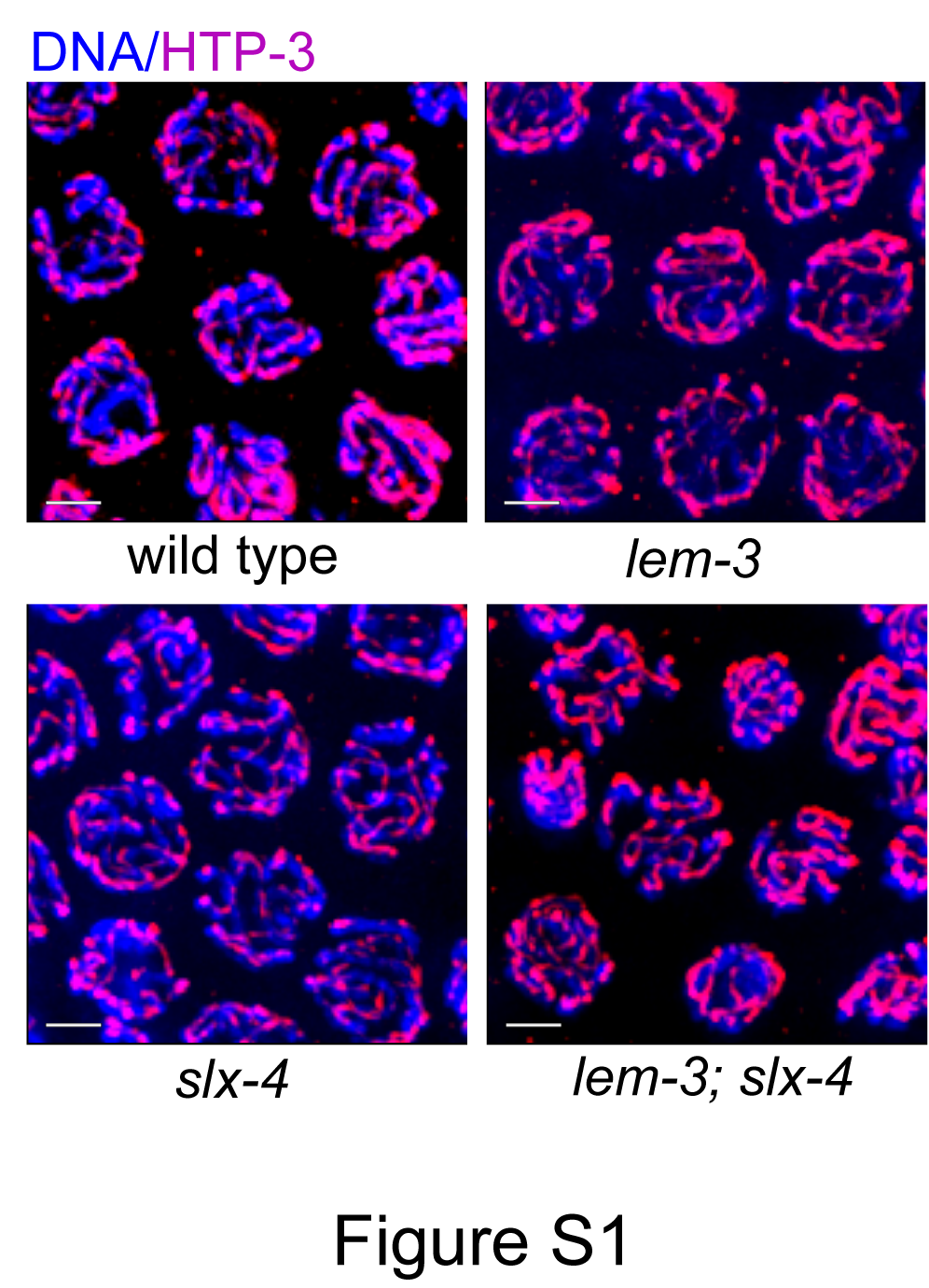

Supplement: S1 Fig — Representative images of pachytene nuclei stained with an antibody recognizing the chromosome axis component HTP-3 (red) and DAPI (blue). Scale bars: 2 μm. (TIF) [file pgen.1007453.s001.tif]

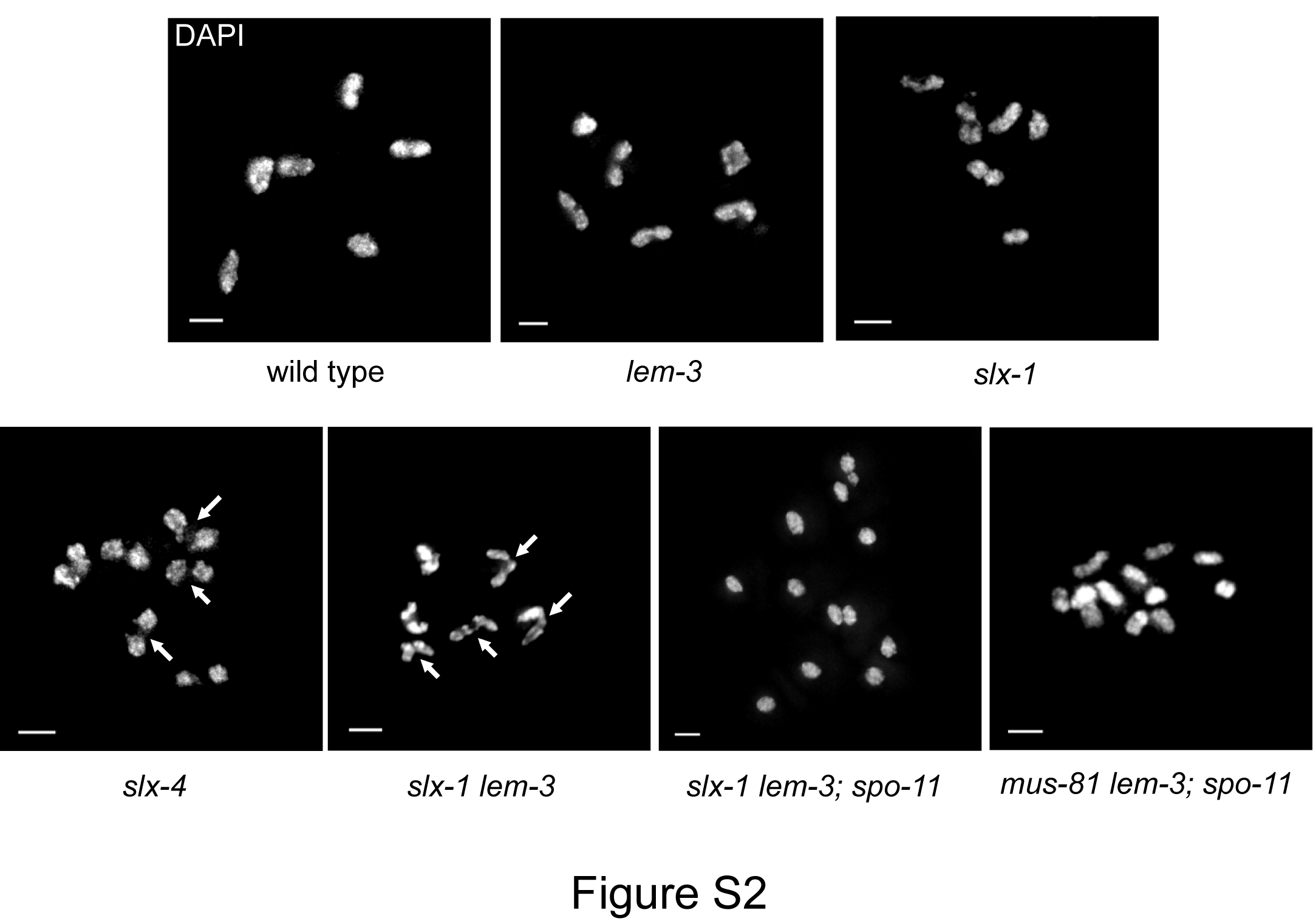

Supplement: S2 Fig — White arrows indicate dissociated bivalents. (TIF) [file pgen.1007453.s002.tif]

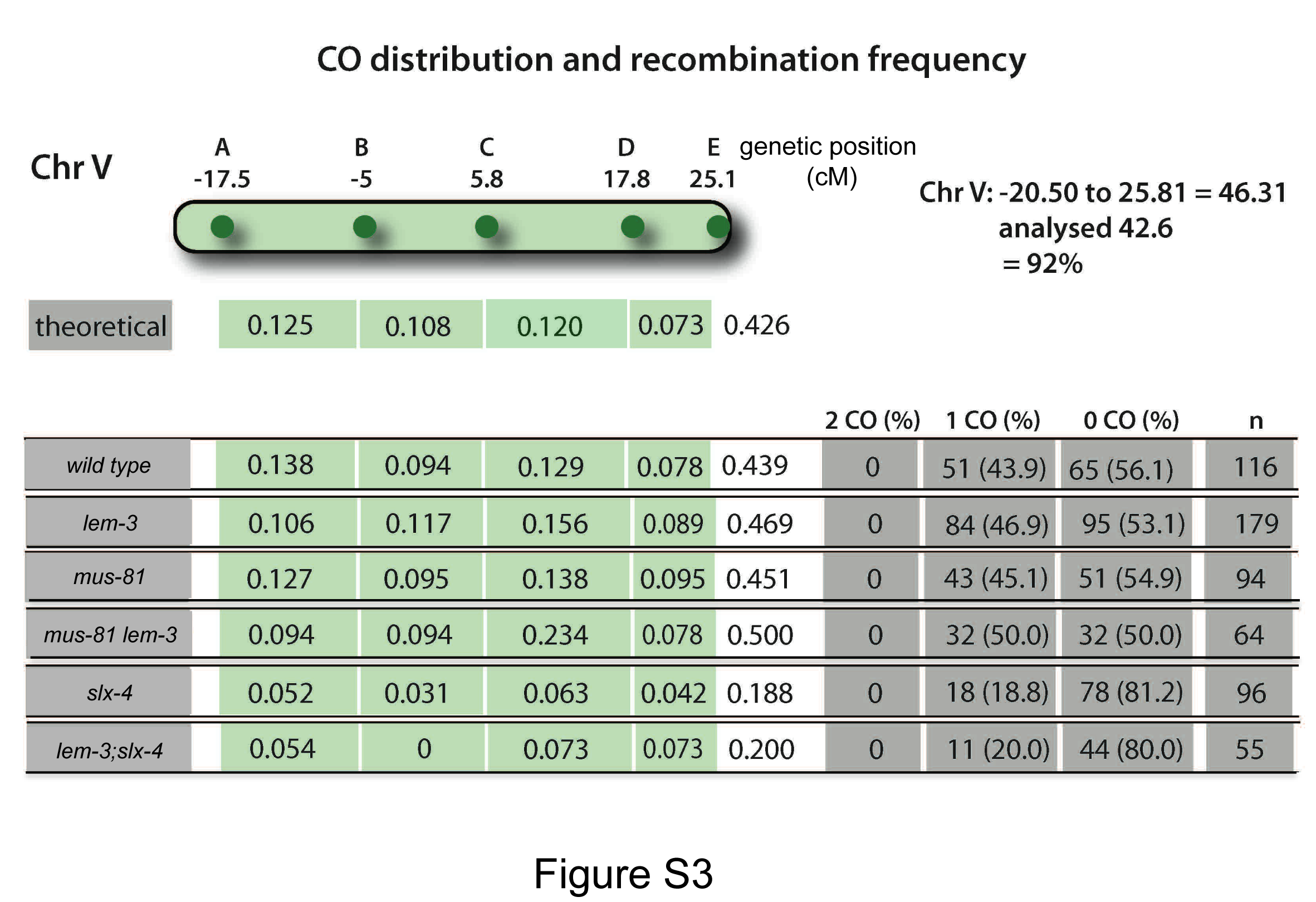

Supplement: S3 Fig — Analysis of crossover frequencies and distribution on chromosome V. The genetic map positions of the five SNPs, which together cover 92% of chromosome V, are indicated. n is the number of cross-progeny scored. The frequency of 2 COs, 1 CO or 0 CO per chromosome is indicated in absolute numbers and as percentage (in brackets). (TIF) [file pgen.1007453.s003.tif]

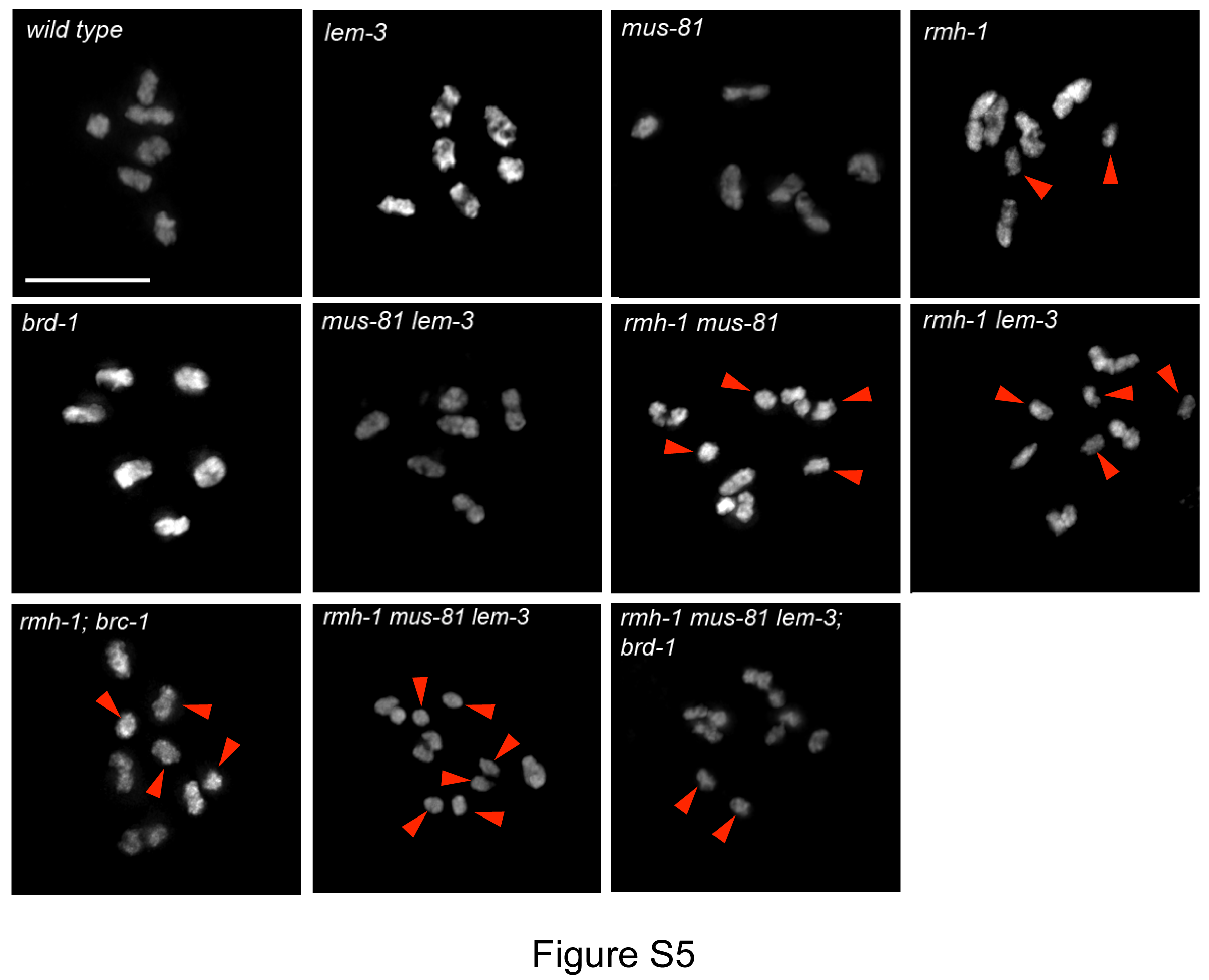

Supplement: S5 Fig — Univalents are indicated by red arrowheads. Scale bar: 5 μm. (TIF) [file pgen.1007453.s005.tif]
