# Supplementary material for: The conserved LEM-3/Ankle1 nuclease is involved in the combinatorial regulation of meiotic recombination repair and chromosome segregation in Caenorhabditis elegans
Source: PLoS Genet. 2018 Jun 7;14(6):e1007453. doi: 10.1371/journal.pgen.1007453 (PMC6007928; doi:10.1371/journal.pgen.1007453)
Supplement: S1 Table — (DOCX) [file pgen.1007453.s010.docx]

**S1 Table. List of strains used in this study.**

| **Genotype** |
| --- |
| *lem-3 (mn155) I* |
| *lem-3 (tm3468) I* |
| *mus-81 (tm1937) I* |
| *slx-1(tm2644) I* |
| *xpf-1(tm2842) II* |
| *rmh-1(jf54) I* |
| *brd-1(gk297) III* |
| *him-18(tm2181)/qC1 III* |
| *mus-81(tm1937) lem-3 (mn155)/hT2 I* |
| *slx-1(tm2644) lem-3 (mn155)/hT2 I* |
| *lem-3 (mn155) I; Hawaii V* |
| *mus-81(tm1937)/hT2 I; Hawaii V* |
| *slx-1(tm2644) I; Hawaii V* |
| *slx-1(tm2644) lem-3 (mn155)/hT2 I; Hawaii V* |
| *mus-81(tm1937) lem-3 (mn155)/hT2 I; Hawaii V* |
| *lem-3 (mn155) I; xpf-1(tm2842) II* |
| *lem-3 (tm3468) I; him-18(tm2181)/qC1 III* |
| *lem-3 (mn155) I; him-18(tm2181)/qC1 III* |
| *rmh-1(jf54) mus-81(tm1937) /hT2 I* |
| *rmh-1(jf54) lem-3(mn155) /hT2 I* |
| *rmh-1(jf54) mus-81(tm1937) lem-3(mn155)/hT2 I* |
| *rmh-1(jf54) lem-3(mn155) I; xpf-1(tm2842) II* |
| *rmh-1(jf54) mus-81(tm1937) I /hT2; xpf-1 (tm2842) II* |
| *lem-3(mn155) I; jfsi38 [gfp::rmh-1 ; cb-unc-119(+)] II* |
| *jfsi38 [gfp::rmh-1 ; cb-unc-119(+)] II.* |
| *lem-3(mn155) I; jfsi38 [gfp::rmh-1 ; cb-unc-119(+)] II* |
| *mus-81 (tm1937) I; jfsi38 [gfp::rmh-1 ; cb-unc-119(+)] II* |
| *mus-81 (tm1937) lem-3(mn155) / hT2 I; jfsi38 [gfp::rmh-1 ; cb-unc-119(+)] II* |
| *odIs57[Ppie-1 mCherry::histoneH2B unc-119(+)], unc-119 (ed3)* |
| *lem-3 (mn155) I; odIs57[Ppie-1 mCherry::histoneH2B unc-119(+)], unc-119 (ed3)* |
| *him-18(*tm2181*)/qC1 III; odIs57[Ppie-1 mCherry::histoneH2B unc-119(+)], unc-119 (ed3)* |
| *lem-3 (mn155) I; him-18(tm2181)/qC1 III; odIs57[Ppie-1 mCherry::histoneH2B unc-119(+)], unc-119 (ed3)* |
| *lem-3 (mn155); GFP::COSA-1* |
| *mus-81 (tm1937)/hT2 I; GFP::COSA-1* |
| *mus-81(tm1937) lem-3 (mn155)/hT2 I; GFP::COSA-1* |
| *him-18(tm2181)/qC1 III; GFP::COSA-1* |
| *lem-3 (mn155) I; him-18(tm2181)/qC1 III; GFP::COSA-1* |
| *cop859 [Plem-3::eGFP::STag:: lem-3::3′UTRlem-3]* |
| *rmh-1(jf54) I mus-81(tm1937) I lem-3(mn155) I; brd-1(gk297) III /hT2* |
| *mus-81(tm1937) lem-3 (mn155)/+ I; spo-11 (me44)/nT1 IV* |
